# In-depth Analysis of the HIV Reservoir Confirms Effectiveness and Safety of Dolutegravir/Lamivudine in a Phase 4 Randomized Controlled Switch Trial (RUMBA)

**DOI:** 10.1093/infdis/jiae405

**Published:** 2024-09-03

**Authors:** Marie-Angélique De Scheerder, Sophie Degroote, Mareva Delporte, Maja Kiselinova, Wim Trypsteen, Lara Vincke, Evelien De Smet, Bram Van Den Eeckhout, Loïc Schrooyen, Maxime Verschoore, Camilla Muccini, Sophie Vanherrewege, Els Caluwe, Stefanie De Buyser, Sarah Gerlo, Evy Blomme, Linos Vandekerckhove

**Affiliations:** Department of Internal Medicine, Ghent University Hospital, Ghent, Belgium; Department of Internal Medicine, Ghent University Hospital, Ghent, Belgium; HIV Cure Research Center, Department of Internal Medicine and Pediatrics, Ghent University, Ghent, Belgium; Department of Internal Medicine, Ghent University Hospital, Ghent, Belgium; HIV Cure Research Center, Department of Internal Medicine and Pediatrics, Ghent University, Ghent, Belgium; Digital PCR Consortium Ghent University, Ghent University, Ghent, Belgium; Department of Internal Medicine, Ghent University Hospital, Ghent, Belgium; HIV Cure Research Center, Department of Internal Medicine and Pediatrics, Ghent University, Ghent, Belgium; HIV Cure Research Center, Department of Internal Medicine and Pediatrics, Ghent University, Ghent, Belgium; Department of Biomolecular Medicine, Ghent University Hospital, Ghent, Belgium; HIV Cure Research Center, Department of Internal Medicine and Pediatrics, Ghent University, Ghent, Belgium; HIV Cure Research Center, Department of Internal Medicine and Pediatrics, Ghent University, Ghent, Belgium; Department of Infectious Diseases, Istituti di Ricovero e Cura a Carattere Scientifico San Raffaele Scientific Institute, Milan, Italy; Department of Internal Medicine, Ghent University Hospital, Ghent, Belgium; Department of Internal Medicine, Ghent University Hospital, Ghent, Belgium; Biostatistics Unit, Faculty of Medicine and Health Sciences, Ghent University, Ghent, Belgium; HIV Cure Research Center, Department of Internal Medicine and Pediatrics, Ghent University, Ghent, Belgium; Department of Biomolecular Medicine, Ghent University Hospital, Ghent, Belgium; HIV Cure Research Center, Department of Internal Medicine and Pediatrics, Ghent University, Ghent, Belgium; Department of Internal Medicine, Ghent University Hospital, Ghent, Belgium; HIV Cure Research Center, Department of Internal Medicine and Pediatrics, Ghent University, Ghent, Belgium

**Keywords:** dual ART, switch randomized controlled trial, HIV-1 reservoir, metabolic health, inflammation

## Abstract

**Background:**

Reducing the number of active compounds for lifelong human immunodeficiency virus (HIV) treatment is of interest, especially to reduce potential long-term side effects. So far, available data assessing viral control support the robustness and safety of 2DR (2-drug regimen) antiretroviral therapy compared to 3DR. However, further in-depth investigations of the viral reservoirs are mandatory to guarantee long-term safety of these regimens regarding stable intact HIV-1 DNA copies, HIV-1 RNA transcripts, and sustained immunological control.

**Methods:**

The RUMBA study is the first prospective randomized controlled trial evaluating the impact of switch from 3DR to 2DR on the viral reservoir. Participants on any stable second-generation integrase strand transfer inhibitor-based 3DR regimen with HIV-1 RNA < 50 copies/mL plasma for at least 3 months were randomized to switch to dolutegravir/lamivudine (DTG/3TC, n = 89) or to switch or stay on bictegravir/emtricitabine/tenofovir alafenamide (B/F/TAF, n = 45). After 48 weeks, virological, immunological, and metabolic parameters were evaluated.

**Results:**

We did not observe a significant difference in change over time in the mean number of intact HIV-1 DNA copies/million CD4^+^ T cells with DTG/3TC compared to B/F/TAF. There was no evidence in this study that switching to DTG/3TC increased the active reservoir by HIV-1 transcription. No significant changes in proinflammatory cytokines or major immune cell subsets were observed. Changes in exhaustion and activation of specific cellular subsets were small and bidirectional. Metabolic outcomes are similar between the treatment regimens.

**Conclusions:**

This study confirms the safety of DTG/3TC compared to B/F/TAF through viral control after in-depth investigations of the intact HIV-1 reservoir, HIV-1 transcription, and inflammatory markers.

**Clinical Trials Registration:**

NCT04553081.

## Teaser

This is the first randomized controlled switch trial confirming the robustness and safety with respect to the viral reservoir, inflammation, and metabolic parameters after switch to dolutegravir/lamivudine (DTG/3TC) compared to bictegravir/emtricitabine/tenofovir alafenamide (B/F/TAF), between baseline and 48 weeks of follow-up.

## Graphical Abstract

**Graphical Abstract:**
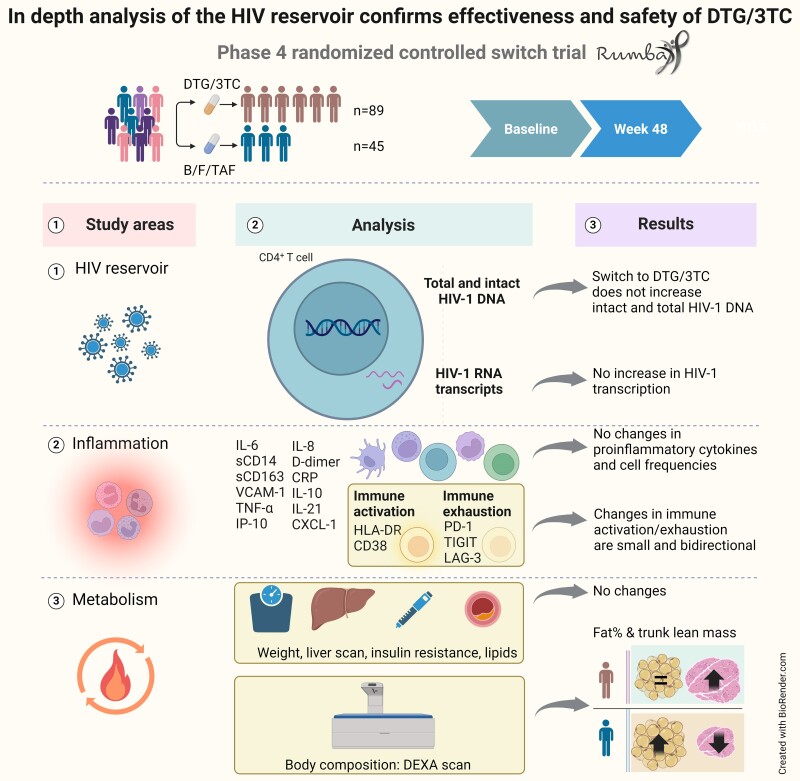
This graphical abstract is also available at Tidbit: https://tidbitapp.io/tidbits/in-depth-analysis-of-the-hiv-reservoir-confirms-effectiveness-and-safety-of-dtg-3tc-in-a-phase-4-randomized-controlled-switch-trial-rumba

Although the availability of well-tolerated, simple and robust combined antiretroviral treatment (ART) has shifted the management of people with human immunodeficiency virus (PWH) from a lethal infectious disease to a chronic condition. HIV-related and non–HIV-related comorbidities persist in this population leading to premature aging and age-related diseases [1]. While sustained virological control and immune reconstitution remain the backbone of HIV management, the introduction of new potent drugs has challenged the concept of triple therapy.

Randomized controlled switch studies [2–4] and ample real-life data have already confirmed the noninferiority in virological outcome of integrase strand transfer inhibitor (INSTI)-based 2-drug regimens (2DR) compared to 3-drug regimens (3DR) in terms of suppressed plasma viremia. However, in-depth investigations looking at the effect on the viral reservoir, measuring the number of latently infected cells, and robust data regarding the beneficial or deleterious effects of 2DR on chronic inflammation, immune activation, or exhaustion are currently lacking.

Although total HIV-1 DNA is mostly assessed as a surrogate marker for the viral reservoir, only replication-competent intact proviruses can reinitiate viral replication upon reactivation from latency. Therefore, total HIV-1 DNA overestimates the reservoir size due to the prevalence of defective proviruses. The intact proviral DNA assay (IPDA) allows differentiation between defective and intact HIV-1 DNA through a multiplexed digital droplet polymerase chain reaction (PCR) reaction and therefore enables an approximation of the active HIV-1 reservoir [5–7].

So far, no prospective clinical trial has used IPDA to compare intact HIV-1 DNA after switching to 2DR. An alternative approach for evaluating the active viral reservoir involves assessing the transcriptional activity of integrated proviruses through cell-associated RNA analysis. Recent developments include not only quantifying full-length transcripts but also identifying transcriptional blocks [8]. The extent to which these blocks can be reversed through simplified treatment remains unclear.

Chronic inflammation is one of the hallmarks of aging and contributes to an increased morbidity in PWH. The SMART trial was the first randomized study to link persistent inflammation and coagulation by measuring interleukin 6 (IL-6) and D-dimers to an increase in non-AIDS–defining illnesses in treated individuals with HIV-1 [9]. Further investigations on a large subset of inflammation, coagulation, and activation markers predicted cardiovascular morbidity and mortality even in those with normal CD4^+^ T cell count [9].

Whether switch from 3DR to 2DR increases the risk of chronic inflammation has been poorly studied. Lower ART adherence and insufficient drug levels in tissues have been linked to excess inflammation and therefore one might hypothesize that switch to 2DR could increase inflammation, immune activation, and exhaustion, and might contribute to excess comorbidity in the long-term. However, to date no study has shown increased inflammation after switching. Therefore, an extensive study looking at the activation/exhaustion state of leukocytes will provide more insights into mechanisms and effects driving inflammation in individuals on ART. Although early and lifelong ART is crucial to reduce the reservoir and inflammation, over time ART regimens have been associated with lipid alterations, diabetes mellitus, and an increase in cardiovascular disease [10, 11]. Whether weight gain associated with more modern ART regimens, mostly combining INSTI and tenofovir alafenamide (TAF), is associated with increased cardiovascular morbidity is still under debate and so far few data are available on reversibility after switching ART regimens [12–15]. Newer techniques are currently being explored to better characterize metabolic changes such as body mass composition parameters and liver steatosis [16, 17].

The RUMBA clinical trial is one of the first head-to-head studies comparing bictegravir/emtricitabine/tenofovir alafenamide (B/F/TAF) and dolutegravir/lamivudine (DTG/3TC). We are the first to present in-depth analyses of the reservoir but also of various inflammatory and metabolic parameters to further enlighten on safety and potential benefits of switching towards 2DR regimens.

## METHODS

### Study Design and End Points

The RUMBA study is a single-center, phase 4, randomized controlled switch study. Participants were recruited at Ghent University Hospital. Participants were on stable second-generation INSTI regimen and virologically suppressed >3 months at the time of inclusion (inclusion and exclusion criteria are in the Supplementary Material). They were randomized 2:1 to either switch to dolutegravir/lamivudine (DTG/3TC) or switch or stay on bictegravir/emtricitabine/tenofovir alafenamide (B/F/TAF).

The study was approved by the Ethics Committee of the Ghent University Hospital (BC-07052) and study participants provided written consent before enrolment and at every amendment. This trial is registered with ClinicalTrials.gov, NCT04553081, and EudraCT No. 2020–000685–42. Amendments to this study were implemented (n = 2) and currently the study will collect data up to 240 weeks of follow-up.

The primary objective is to compare DTG/3TC to B/F/TAF at week 48 after switch by measuring intact HIV-1 DNA copies in CD4^+^ T cells. Secondary and exploratory outcomes were chosen to further confirm the safety of DTG/3TC in terms of virological and immunological control, and potentially show an advantage of DTG/3TC on metabolic health. An overview of all clinical, laboratory, and treatment data collected and detailed methods on collection and analyses can be found in Supplementary Table 1.

### Cross-Subtype IPDA With Total HIV-1 DNA

The recently published cross-subtype IPDA, originally established on the 2-color Bio-Rad system (QX200), was modified for the Qiacuity 5-color system, with the addition of the total HIV-1 DNA assay (targeting RU5). The RU5, long terminal repeat (LTR)-gag, 5′ pol, and env probes were linked to ROX, Cy5, FAM, and HEX fluorophores, respectively (Supplementary Table 2). Primer/probe concentrations were used as described by Cassidy et al [18] (Supplementary Table 3). The digital PCR program started with 2 minutes at 95°C, followed by 65 cycles of 94°C for 30 seconds and 60°C for 60 seconds. Samples were run in triplicate with positive controls (J-Lat genomic DNA [gDNA] 413 spiked into HIV-1–negative CD4^+^ T cell gDNA) and negative controls (nuclease-free water).

The *RPP30* duplex assay was performed according to Bruner et al [5] for normalization based on cell input. In addition, the DNA shearing index was used to correct the intact HIV-1 DNA copy number.

### Statistical Analysis

Block randomization with fixed block size was applied with a 2:1 allocation ratio (2DR:3DR). REDCap, an electronic web-based data capture system, was used for data collection [19]. Analysis was conducted using R version 4.2.2 [20]. Ordinary linear regression models were fitted to compare the mean change from baseline between treatment groups. Most end points were log-transformed to reduce skewness. No adjustment for multiple testing has been performed, given the large number of exploratory end points. Missing data were handled using multiple imputation. Results from complete case analyses can be found in Supplementary Material 2. Unless explicitly mentioned, point estimates and 95% confidence intervals (CIs) described in the results section refer to the intention-to-treat exposed (ITT-E) analysis after multiples of missing data.

## RESULTS

### Demographics and Baseline Characteristics

Between June 2020 and August 2021, 134 participants were randomized, of which 130 participants were exposed to the treatment and included in the ITT-E analysis set (DTG/3TC, n = 87 and B/F/TAF, n = 43) (Supplementary Figure 1, consort diagram). Baseline characteristics were well balanced between treatment groups, except for a significant difference in body mass index (BMI) and peak viral load between the groups (Table 1). Between initiation and week 48, 4 participants discontinued due to adverse events (B/F/TAF = 1, DTG/3TC = 3); none were linked to virological failure (VF) (Supplementary Table 4). Virological success was high in both arms, resulting in 92.5% (B/F/TAF) and 98.8% (DTG/3TC) of participants with a viral load <50 copies/mL at week 24 and 98% of participants <50 copies/mL in both arms at week 48. We assessed adherence based on pill counts, patient reporting, and viral control. Adherence below 100% was not associated with risk of VF.

**Table 1.** Baseline Characteristics of Randomized Participants

Characteristic	Total (n = 134)		B/F/TAF, 3-Drug Regimen (n = 45)		DTG/3TC, 2-Drug Regimen (n = 89)	
Intention-to-treat exposed population	n = 130		n = 43		n = 87	
Sex, M/F	118/12		39/4		79/8	
Ethnicity, European/African/other	102/14/14		32/5/6		70/9/8	
Smoking status, never/exsmoker/current smoker	57/24/49		24/6/13		33/18/36	
Subtype, B/non-B	86/44		26/17		60/27	
Age, y, median (IQR)	47	(37–55)	46	(38–52)	48	(40–56)
CD4 at screening, cells/µL, median (IQR)	689	(548–926)	603	(519–765)	690	(525–926)
CD4 nadir, cells/µL, median (IQR)	302	(200–459)	256	(130–332)	309	(155–449)
CD4/CD8 ratio, median (IQR)	1.02	(0.7–1.38)	1.02	(0.78–1.32)	0.96	(0.64–1.38)
Peak viral load, copies/mL plasma, median (IQR)	97 647	(26 380–332 504)	80 670	(17 951–193 505)	190 546	(57 544–436 516)
Baseline regimen, DTG/ABC/3TC, B/F/TAF, DTG + FTC/TAF	49/80/1		22/21/0		27/59/1	
Time on ART, y, median (IQR)	7.2	(4.6–10.8)	6	(4.35–8.95)	8.6	(5.2–11.5)
Time from start ART to undetectable viral load, y, median (IQR)	0.3	(0.2–0.4)	0.3	(0.2–0.7)	0.3	(0.2–0.4)
Total HIV-1 DNA, copies/10^6^ CD4^+^ T cells, median (IQR)	485	(228–1110)	414	(163–1410)	503	(254–1044)
Intact HIV-1 DNA, copies/10^6^ CD4^+^ T cells, median (IQR)	44	(12–109)	58	(12–191)	43	(10–95)
Ratio intact/total HIV-1 DNA, %, median (IQR)	5.8	(1.8–10.3)	8.2	(3.9–12.6)	4.8	(1.5–8.4)
BMI, kg/m², median (IQR)	25	(23–28)	25	(22–26)	26	(23–28)

Data are No. except where indicated.

Abbreviations: 3TC, lamivudine; ABC, abacavir; ART, antiretroviral therapy; B, bictegravir; BMI, body mass index; DTG, dolutegravir; F/FTC, emtricitabine; HIV, human immunodeficiency virus; IQR, interquartile range; TAF, tenofovir alafenamide.

### Switch to DTG/3TC Does Not Seem to Impact the Size or the Activity of the Viral Reservoir

We observed a mean decrease in total HIV-1 DNA copies per million CD4^+^ T cells at week 48 from baseline, which was significant in B/F/TAF (−30%; 95% CI [−49%; −5%]), but not significant in DTG/3TC (−14%; 95% CI [−32%; +9%]). However, between the arms, we could not find a significant difference in mean relative decrease (Table 2).

**Table 2.** Virological End Points

				ITT-E (n = 130)			B/F/TAF, 3DR (n = 43)			DTG/3TC, 2DR (n = 87)		
				Treatment Ratio 3DR/2DR			Week 48 Visit to Baseline Ratio			Week 48 Visit to Baseline Ratio		
End Point	Population	Model	n	Estimate	95% LCL	95% UCL	Estimate	95% LCL	95% UCL	Estimate	95% LCL	95% UCL
Total HIV-1 DNA	ITT-E	MI	130	0.81	0.57	1.15	0.70	0.51	0.95	0.86	0.68	1.09
Total HIV-1 DNA	PP	MI	120	0.79	0.56	1.10	0.69	0.53	0.91	0.88	0.73	1.06
Total HIV-1 DNA^a^	ITT-E	MI	130	0.79	0.55	1.14	0.48	0.25	0.93	0.61	0.33	1.14
Intact HIV-1 DNA	ITT-E	MI	130	1.30	0.80	2.11	1.43	1.00	2.05	1.10	0.82	1.49
Intact HIV-1 DNA	PP	MI	120	1.30	0.82	2.05	1.35	0.93	1.96	1.04	0.80	1.34
Intact HIV-1 DNA^a^	ITT-E	MI	130	1.29	0.78	2.13	0.68	0.30	1.54	0.53	0.25	1.13
Intact HIV-1 DNA^b^	ITT-E	MI	130	1.40	0.88	2.22	1.50	1.06	2.14	1.08	0.81	1.43
Intact HIV-1 DNA without DSI	ITT-E	MI	130	1.31	0.82	2.10	1.49	1.03	2.15	1.13	0.86	1.50
Intact HIV-1 DNA without DSI	PP	MI	120	1.32	0.84	2.09	1.40	0.97	2.03	1.06	0.82	1.37
Intact HIV-1 DNA without DSI^a^	ITT-E	MI	130	1.31	0.80	2.13	0.72	0.32	1.63	0.55	0.26	1.17
Intact HIV-1 DNA without DSI^b^	ITT-E	MI	130	1.42	0.90	2.24	1.57	1.09	2.25	1.11	0.84	1.45
Intact HIV-1 DNA DTG/ABC/3TC^c^	ITT-E	MI	49	1.63	0.86	3.10	1.51	0.93	2.45	0.93	0.61	1.41
Intact HIV-1 DNA B/F/TAF^d^	ITT-E	MI	80	1.07	0.52	2.19	1.34	0.77	2.31	1.25	0.85	1.85
TAR copies/µg RNA	ITT-E	CCA	74	1.13	0.83	1.55	1.14	0.88	1.47	1.00	0.85	1.18
Long LTR copies/µg RNA	ITT-E	CCA	74	1.11	0.78	1.56	0.97	0.73	1.29	0.87	0.73	1.05
Pol copies/µg RNA	ITT-E	CCA	74	0.83	0.55	1.25	0.81	0.58	1.14	0.98	0.79	1.22
TAR copies normalized	ITT-E	CCA	74	1.42	0.91	2.21	1.73	1.19	2.50	1.22	0.96	1.54
Long LTR copies normalized	ITT-E	CCA	74	0.96	0.68	1.37	1.15	0.85	1.54	1.19	0.98	1.43
Pol copies normalized	ITT-E	CCA	74	0.87	0.57	1.32	0.94	0.66	1.33	1.08	0.87	1.35

Virological changes after 48 weeks, corrected for baseline response value, CD4 nadir and time on ART. The estimated geometric mean ratio of the groups’ (3DR/2DR) relative change from baseline (week 48/D1) is reported with 95% CI, calculated using an ordinary linear regression model applied to change from baseline in natural log-transformed imputed data (MICE, number of multiple imputations = 50). Analysis was performed on both the ITT-E and PP analysis set. Model for missing data handling: MI and CCA. RNA transcripts were normalized by dividing copies HIV RNA by the geometric mean of 3–4 reference genes per patient (*B2M*, *HPRT1*, and *YWHAZ* for TAR, *B2M* + *HPRT* + *YWHAZ* + *TBP* for the others). Tat-rev multiple-spliced transcripts (surrogate for productive infection) were undetectable in 91% of the samples.

Abbreviations: 2DR, 2-drug regimen; 3DR, 3-drug regimen; 3TC, lamivudine; ABC, abacavir; ART, antiretroviral therapy; B, bictegravir; CCA, complete case analysis; CI, confidence interval; DSI, DNA shearing index; DTG, dolutegravir; F, emtricitabine; HIV, human immunodeficiency virus; ITT-E, intention to treat exposed; LCL, lower confidence interval limit; LTR, long terminal repeat; MI, multiple imputation; PP, per protocol; TAF, tenofovir alafenamide; TAR, trans-activation response element; UCL, upper confidence interval limit.

^a^Additionally corrected for baseline regimen and peak viral load.

^b^Additionally corrected for total HIV DNA at baseline.

^c^Analysis for the subgroup with DTG/ABC/3TC as baseline regimen.

^d^Analysis for the subgroup with B/F/TAF as baseline regimen.

**Figure 1.**
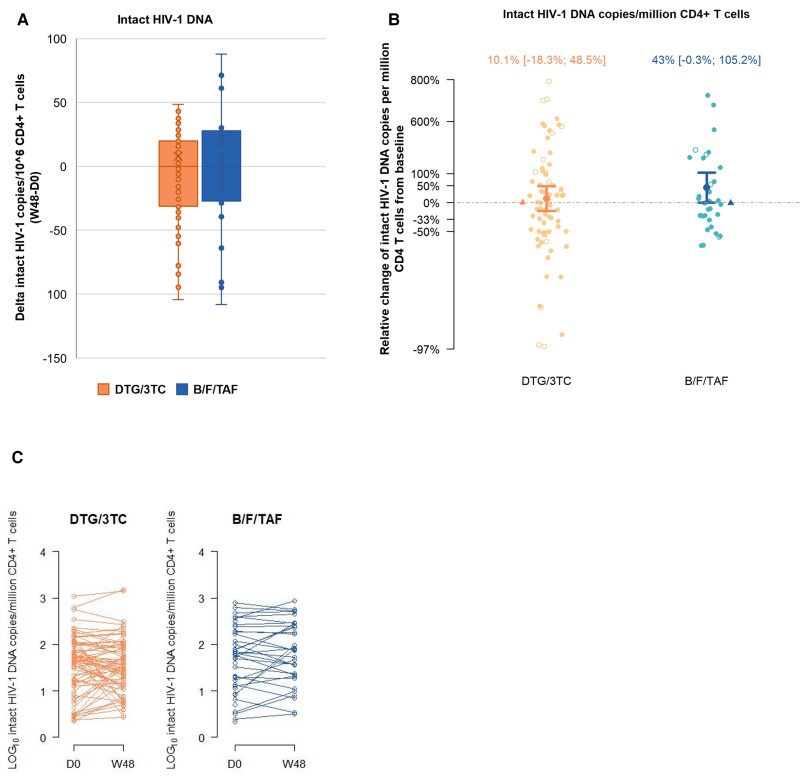
Intact HIV-1 DNA. *A*, The absolute change in the number of intact HIV-1 DNA copies between week 48 and baseline, after correction for DNA shearing (median and IQR are reported; whiskers represent 1.5 IQR). Graph based on complete cases B/F/TAF = 33, DTG/3TC = 72, with outliers >1.5 IQR excluded (B/F/TAF = 5, DTG/3TC = 6). *B*, The relative change of intact HIV-1 DNA copies per million CD4 T cells from baseline, after correction for DNA shearing. Estimated mean values are reported with upper and lower confidence interval limit estimates, calculated using an ordinary linear regression model applied to change from baseline in natural log-transformed imputed data. Multiple imputations analysis in the intention-to-treat exposed set (n = 130) adjusted for baseline response value, CD4 nadir, and time on antiretroviral therapy. The triangle symbol represents the observed median change. Closed dots contain measurements above the LOD; open dots contain measurements below the LOD, replaced by the LOD/2, adjusted for cell input and DNA shearing index. *C*, Dynamics of intact HIV-1 DNA in the 2 treatment arms expressed as log_10_ copies/million CD4^+^ T cells at baseline (day 0) and after 48 weeks. Abbreviations: 3TC, lamivudine; B, bictegravir; DTG, dolutegravir; F, emtricitabine; HIV-1, human immunodeficiency virus-1; IQR, interquartile range; LOD, limit of detection; TAF, tenofovir alafenamide.

The intact fraction represented 5.8% of the total HIV-1 DNA in the overall population at baseline (Table 1). The observed median absolute change over time in the number of intact HIV-1 DNA copies was similar in both treatment arms and was close to zero (Figure 1*A*). After adjustment for covariates and multiple imputations, the observed increase in estimated mean number of intact HIV DNA copies per million CD4 T cells from baseline to week 48 was not significant, at 10% (95% CI, .82–1.49) and 43% (95% CI, 1.00–2.05) with 2DR and 3DR, respectively (Figure 1*B*). The dynamics were similar in both arms (Figure 1*C*); we did not observe a significant mean relative difference in the primary end point, neither over time, nor between treatment arms in both the ITT-E and per protocol analysis (Table 2).

Also, in subgroups based on baseline regimen before start of the study, in models without correction for DNA shearing, or with additional adjustment for baseline regimen, peak viral load, or total HIV DNA, we observed no evidence for a clinically meaningful difference between the treatment arms for the primary end point (Table 2). In participants with subtype B virus (n = 74), we did not see a difference in the number of transcriptionally initiated (trans-activation response element [TAR], binding site of TAT), elongated, and unspliced HIV-1 RNA transcript copies between the treatment arms (Table 2).

### Changes in Immune Activation and Exhaustion Are Small and Bidirectional

We could not find a significant difference in mean relative change between treatment arms from baseline to week 48 in the quantified inflammatory markers such as IL-6, sCD14, sCD163, vascular cell adhesion molecule-1 (VCAM-1), tumor necrosis factor-α (TNF-α), inducible protein-10 (IP-10), IL-8, and D-dimer. Furthermore, we did not observe changes in CD4/CD8 ratio and the percentages of relevant immune cells (CD4 T, CD8 T, B, NK, NKT cells, monocytes, and dendritic cells) (Supplementary Table 5).

Next, we looked at the activation/exhaustion status of all immune cells. In this large dataset, we only found a few differences. With B/F/TAF, activation (HLA-DR, CD38) was significantly higher of CD4 effector memory T cells (T_EM_; 25%; 95% CI, 1.10–1.43), CD8NKT (58%; 95% CI, 1.13–2.23), and γδ T cells (23%; 95% CI, 1.04–1.46) (Table 3). In this treatment group, exhaustion markers also increased in the γδ T cell fraction (programmed cell death protein 1 [PD-1] 21%; 95% CI, 1.09–1.33 and TIGIT 56%; 95% CI, 1.15–2.11), while in the DTG/3TC group, we saw increased expression of exhaustion markers in CD8 (PD-1 6%; 95% CI, 1.01–1.12) and CD8 T_EMRA_ cells (TIGIT 5%; 95% CI, 1.02–1.09).

**Table 3.** Immune Activation and Exhaustion Markers

	ITT-E (n = 130)			B/F/TAF, 3DR (n = 43)			DTG/3TC, 2DR (n = 87)		
	Treatment Ratio 3DR/2DR			Week 48 Visit to Baseline Ratio			Week 48 Visit to Baseline Ratio		
Marker	Estimate	95% LCL	95% UCL	Estimate	95% LCL	95% UCL	Estimate	95% LCL	95% UCL
CD4 T									
CD4 T	0.94	.82	1.07	0.93	.82	1.04	0.99	.91	1.07
HLA-DR	1.10	.98	1.22	1.14	1.02	1.26	1.04	.97	1.11
CD38^a^	1.17	−.99	3.33	2.07	.04	4.11	0.90	−.51	2.31
TIGIT	0.97	.92	1.02	1.02	.97	1.07	1.05	1.01	1.09
LAG-3	0.86	.67	1.09	0.99	.79	1.24	1.16	.99	1.36
PD-1	0.99	.90	1.08	1.02	.93	1.11	1.03	.97	1.10
T_EM_	1.05	.95	1.16	0.99	.9	1.09	0.94	.88	1.01
T_EM_ HLA-DR	1.22	***1.06***	***1.41***	1.25	**1.10**	**1.43**	1.03	.94	1.12
T_EM_ HLA-DR^+^PD-1^+^CD38^−^	1.16	***1.01***	***1.34***	1.11	.97	1.28	0.96	.87	1.06
T_EMRA_	1.19	.92	1.54	1.09	.84	1.41	0.92	.77	1.09
T_EMRA_ PD-1	0.79	***.64***	***.96***	0.86	.70	1.06	1.10	.97	1.24
CD8 T									
CD8 T	0.95	.84	1.08	0.93	.83	1.04	0.97	.91	1.05
HLA-DR	1.09	.91	1.30	1.23	1.04	1.47	1.14	1.01	1.28
CD38	1.08	.97	1.21	1.13	1.01	1.26	1.04	.97	1.12
TIGIT	0.98	.93	1.04	1.02	.97	1.08	1.04	1.00	1.08
LAG-3	0.86	.69	1.06	1.10	.89	1.36	1.28	1.11	1.49
PD-1	0.94	.88	1.00	1.02	.95	1.09	1.09	1.04	1.14
PD-1^+^HLA-DR^−^CD38^−^	0.91	***.84***	***.97***	0.96	.90	1.03	1.06	**1.01**	**1.12**
CD27^+^ T_EMRA_	1.02	.92	1.12	1.01	.91	1.11	0.99	.93	1.05
PD-1	0.93	***.87***	***.99***	0.98	.92	1.05	1.06	**1.01**	**1.11**
PD-1^+^ HLA-DR^−^CD38^−^	0.87	***.77***	***.99***	0.98	.86	1.11	1.12	**1.03**	**1.22**
TIGIT	0.93	***.89***	***.99***	0.99	.94	1.04	1.05	**1.02**	**1.09**
CD27^−^ T_EMRA_	1.05	.91	1.20	0.96	.84	1.10	0.92	.85	1.00
PD-1^+^HLA-DR^−^CD38^−^	0.84	*.73*	*.98*	0.92	.80	1.07	1.09	1.00	1.20
CD25^+^CD45RA^+^	1.13	.94	1.36	1.17	.97	1.42	1.04	.91	1.18
PD-1	0.88	.80	.98	0.95	.85	1.05	1.07	1.00	1.15
CD8 NKT									
CD8 NKT	0.98	.88	1.09	0.95	.85	1.06	0.96	.89	1.04
HLA-DR	1.61	***1.13***	***2.29***	1.58	**1.13**	**2.23**	0.99	.78	1.24
γδ T									
γδ T	0.90	*.81*	*.99*	0.91	.83	1.00	1.02	.95	1.09
CD8^+^	1.10	*1.02*	*1.17*	1.06	.99	1.14	0.97	.93	1.02
CD4^−^CD8^−^	0.90	*.84*	*.97*	0.94	.87	1.01	1.04	.99	1.09
HLA-DR	1.13	.93	1.37	1.26	1.05	1.51	1.11	.98	1.27
CD38	1.26	***1.06***	***1.51***	1.23	**1.04**	**1.46**	0.98	.87	1.09
PD-1	1.36	***1.22***	***1.51***	1.21	**1.09**	**1.33**	0.89	.83	.95
LAG-3, MFI^a^	−3.42	−13.64	6.80	−0.27	−9.62	9.09	3.15	−3.13	9.43
TIGIT, MFI	1.85	***1.36***	***2.51***	1.56	**1.15**	**2.11**	0.84	.69	1.03

Immunological changes after 48 weeks, corrected for age category (≤50 y, > 50 y), CD4/CD8 ratio, smoking status, and baseline response value in the ITT-E population. Estimated ratios are reported with upper and lower confidence interval limit estimates, calculated using an ordinary linear regression model applied to change from baseline in natural log-transformed imputed data. The confidence intervals highlighted in bold and italic do not contain the null value for the treatment ratios. Confidence intervals for the week 48 visit to baseline ratios that do not contain the null value are in bold.

Abbreviations: 2DR, 2-drug regimen; 3DR, 3-drug regimen; 3TC, lamivudine; B, bictegravir; DTG, dolutegravir; F, emtricitabine; ITT-E, intention to treat exposed; LCL, lower confidence interval limit; MFI, median fluorescence intensity; TAF, TAF, tenofovir alafenamide; UCL, upper confidence interval limit.

^a^For untransformed end points, the estimated arithmetic mean difference in (absolute) change from baseline between groups is reported with 95% confidence interval. The gating strategy can be found in Supplementary Figure 2.

### Differences in Body Composition Observed After Switch to DTG/3TC

**Figure 2.**
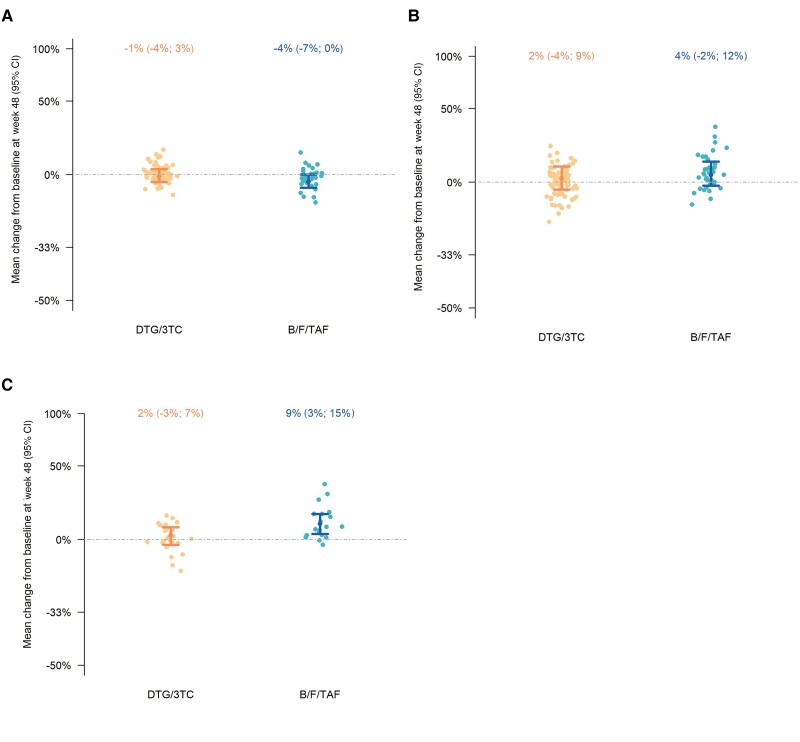
Differences in body composition. The relative change of trunk lean mass (*A*) and fat percentage (*B*) from baseline. *C*, The relative change of fat percentage in the subgroup of participants with no previous TAF exposure. Multiple imputations analysis in the intention-to-treat exposed set (n = 130) adjusted for baseline response value, baseline regimen, and baseline BMI (*A* and *B*) and for baseline response value and baseline BMI (*C*). Abbreviations: BMI, body mass index; CI, confidence interval; TAF, tenofovir alafenamide.

Concerning all secondary and exploratory metabolic end points, similar mean relative changes from baseline at week 48 were found (Supplementary Table 6). Overall, we observed a mean decrease in trunk lean mass from baseline at week 48 (estimated at −4% with B/F/TAF and −1% with DTG/3TC; Figure 2*A*) and a mean increase in fat percentage from baseline at week 48 (estimated at +4% with B/F/TAF and +2% with DTG/3TC; Figure 2*B*). For the subgroup of participants who did not have previous TAF exposure (37.7%) the estimated mean increase in fat percentage from baseline at week 48 was significantly higher when switching to B/F/TAF (+9%; 95% CI [+3%; +15%]) compared to DTG/3TC (+2%; 95% CI [−3% ;+7%]; Figure 2*C*).

## DISCUSSION

This is the first randomized controlled switch trial comparing DTG/3TC to B/F/TAF with respect to viral reservoir, inflammation, and metabolic parameters between baseline and 48 weeks of follow-up.

**Virological outcomes:** In accordance with previous studies, switch to DTG/3TC was safe in terms of virological suppression at week 48 [2, 4]. Our in-depth investigations of the HIV-1 viral reservoir after switch to DTG/3TC further strengthen these findings, providing no indication of a clinically important difference in relative change from baseline at week 48 in both intact and total HIV-1 DNA copies. Total HIV-1 DNA declined in both arms at week 48 from baseline, with a significant decline in B/F/TAF, but nonsignificant in DTG/3TC. The CIs of these mean declines are quite wide, resulting in no observed significant difference between the arms. It is well known that total HIV decays slowly during suppressive ART depending on the time on ART [21], which was well balanced between the 2 groups. In addition, clones carrying replication-competent HIV-1 wax and wane [22] and our data suggest that reducing the number of active compounds with a robust INSTI backbone does not favor the expansion of clones harboring intact viral sequences. The nonsignificant increase in the fraction of intact HIV-1 DNA in both arms is probably reflecting the natural variation of intact clones over time and the dynamics of the intact reservoir. Final conclusions on the presence of intact and defective clones can only be made after sequencing the full-length HIV genome, or the integration sites, which is very labor intensive and these platforms are not suitable for implementation in larger clinical trials yet. In addition, about half of the participants had been on ART for less than 7 years, a period in which more dynamic changes are still observed, according to Peluso et al who followed participants for a median of 7.3 years [23]. One study reported an increase in the viral reservoir as measured by quantitative viral outgrowth assay [24]. Gandhi et al reported a decrease in intact HIV DNA between a median of 7.1, 10.8, and 12.6 years after ART initiation [25]. Furthermore, Altar et al. also reported a faster decay in intact versus defective proviruses at time points 7 years apart [26]. The contrasting result in these studies can potentially be explained by different periods and methods of evaluation. The dynamics of intact HIV-1 DNA also show that there is quite a large variability at baseline and over time for this measure, stressing the importance of a large sample size. Our results are in line with findings of a smaller retrospective study, where no significant change in total and intact HIV-1 DNA was observed in the DTG/3TC group (n = 23) over 48 weeks [27]. Furthermore, we are the first to explore the transcriptional activity of the viral reservoir in a switch trial, going beyond the full-length, unspliced HIV-RNA measurement. In line with the TRIDUAL trial and extended here with data on initiated, elongated, and spliced HIV-RNA transcripts, we confirm that simplification to DTG/3TC does not increase HIV-1 transcription.

**Immunological outcomes:** Our data are in line with previous publications on inflammatory markers after simplification to 2DR, and mostly DTG/3TC. Although the SALSA and TANGO studies showed reduced levels of sCD14 in favor of 2DR, TANGO also reported lesser increase in IL-6 levels in 3DR versus 2DR at some time points, questioning the relevance of these individual markers and their contribution to clinical outcomes and highlighting the multifactorial pathways of inflammation and immune activation [2, 4]. Like our study, recent TRIDUAL and DEBATE results did not observe changes in these markers at week 48 [28, 29]. Those 2 studies also reported no changes in activation and exhaustion in CD4 and CD8 T cells after 12 months and up to 96 weeks, respectively. In our study, we observed slightly higher levels of exhausted CD8 T cells with DTG/3TC, a trend that also was observed after 6 months in the DEBATE study but attenuated after 12 months [29]. Whether the increase in exhaustion in our study persists over time and if this change is clinically relevant, will be explored at follow-up time points.

We are the first to report on an extensive panel of immune cell subsets and their activation/exhaustion state, going beyond the previously reported data. Overall, these data needs to be interpreted with caution due to the small and bidirectional differences, often with broad CI. Follow-up data of the RUMBA study will assess whether these observations persist over time and whether they can be linked to an increased viral transcription, clonal expansion of intact HIV-1 DNA copies, or inflammatory parameters, which was not observed at this time point. Recently, it has been shown that TIGIT expression tracks with inflammatory activity of γδ T cells during ART suppression [30]. Because our data suggest that activation/exhaustion is more present in γδ T cells in B/F/TAF, more extensive research on these subsets and longitudinal follow-up will be of interest for future research. It further questions whether ART toxicity should be taken into account as a contributing factor rather than suboptimal viral control [31].

**Metabolic outcomes:** Metabolic outcomes were broadly similar between the arms and this confirms results of previous randomized controlled trials (RCTs) [2, 4, 13]. However, 2 body composition measures (trunk lean mass and fat percentage) seemed to evolve in a favorable way with DTG/3TC. We conducted the first RCT to include body composition measurements to assess metabolic end points in a switch study to DTG/3TC. Previous studies looking at body composition after switch to 2DR did not find significant differences between protease inhibitor (PI)-based and INSTI-based regimens [32, 33]. The Advance trial showed excess weight gain mostly after initiating DTG/FTC/TAF compared to DTG/TDF/FTC and TDF/FTC/EFV, which was associated with limb and trunk fat increase but also an increase in lean mass [12]. Recently, follow-up data on switching from the TAF regimen to TDF showed improvement in weight, BMI, cholesterol, and glucose measurements; unfortunately, no body composition measurements after switch to TDF have been presented so far [12]. In our study, all participants were treated with second-generation INSTI at baseline and most were already exposed to TAF (62.3%). In participants switching to TAF we did see a significant change in fat percentage, although this was not associated with differences in weight gain between groups, underlining the value of body composition markers in evaluating metabolic health [34]. Longitudinal follow-up will help to provide more insight into the metabolic effects of therapy switch and the impact on comorbidity and cardiovascular risk in this study population.

Limitations of the study include that sample size calculation for the primary end point was challenging and was based on the IPDA method with limited data from the literature at the time of preparation of the study. Prior to statistical analysis, power calculations were performed with respect to the primary end point, based on the publications of Bruner et al and Dragoni et al [5, 27]. Our sample size of 134 participants would achieve limited power to conclude noninferiority with a noninferiority margin of 12% of 2DR compared to 3DR at the 2.5% 1-sided significance level with respect to mean number of intact HIV-1 copies per million CD4^+^ T cells at week 48. Therefore, noninferiority assumptions and null hypothesis for this type of end point should be carefully determined based on accumulation of data, and our findings should be confirmed in larger RCTs. Results should be considered hypothesis generating, hence, no *P* values are reported. All virological and immunological assays were conducted on blood cells and plasma. We did not have access to tissue samples to exclude increased local viral replication, which might trigger cell activation; however, such potential changes in tissues would eventually be reflected in the periphery (blood). Randomization was not stratified according to BMI and we noted differences at baseline. However, all metabolic outcomes were adjusted for BMI category.

## CONCLUSION

In-depth investigations of the viral reservoir in the RUMBA clinical trial confirms the robustness and safety after switch to DTG/3TC compared to B/F/TAF. Switch to DTG/3TC does not seem to increase the HIV-1 reservoir in terms of intact and total HIV-1 DNA copies, nor does it influence viral RNA transcription or the production of proinflammatory cytokines. Although we do see some differences in immune activation/exhaustion in several cell subsets, most of these changes are small and bidirectional, without a clear clinical impact and potential mechanistic drivers should be further explored. Metabolic outcomes at week 48 are similar between the treatment regimens and only showed minor differences potentially in favor of DTG/3TC. Further longitudinal data (week 144 and week 240) are being collected to investigate whether these trends can be confirmed over the longer term.

## Supplementary Data

Supplementary materials are available at *The Journal of Infectious Diseases* online (http://jid.oxfordjournals.org/). Supplementary materials consist of data provided by the author that are published to benefit the reader. The posted materials are not copyedited. The contents of all supplementary data are the sole responsibility of the authors. Questions or messages regarding errors should be addressed to the author.

## Supplementary Material

jiae405_Supplementary_Data

## Notes

***Author contributions.*** M. A. D. S. and L. V. D. K. contributed conceptualization and acquired funding. E. B., S. D., S. D. B., M. D., W. T., B. V. D. E., and S. G. contributed methodology. M. A. D. S., L. V., E. B., E. D. S., M. V., L. S., L. V., and C. M. performed investigation. E. B., S. D., S. D. B., M. D., W. T., and M. V. performed formal analysis. M. A. D. S., L. V. D. K., and E. B. performed supervision. M. A. D. S., L. V. D. K., M. K., S. V. H., and E. C. contributed resources. E. B., S. D., and M. A. D. S. wrote the original draft. E. B., S. D., M. A. D. S., L. V. D. K., S. G., S. D. B., and M. D. reviewed and edited the manuscript.

***Disclaimer***. ViiV Healthcare was not involved in the study design, data analysis, or writing of the manuscript.

***Financial support*.** This work was supported by ViiV Healthcare (KW/2042/CIZ/001/002).
