# Supplementary material for: In-depth Analysis of the HIV Reservoir Confirms Effectiveness and Safety of Dolutegravir/Lamivudine in a Phase 4 Randomized Controlled Switch Trial (RUMBA)
Source: J Infect Dis. 2024 Sep 3;231(1):e91–e100. doi: 10.1093/infdis/jiae405 (PMC11793038; doi:10.1093/infdis/jiae405)
Supplement: jiae405_Supplementary_Data [file jiae405_supplementary_data.zip › Annex_1_SAP_Rumba_study_v1.0_20230408.pdf]

2DR study: Statistical analysis plan

Administrative information

TRIAL FULL TITLE	Virological and immunological assessment in HIV positive participants on 2DR versus 3DR in a prospective randomized controlled switch trial.
SAP VERSION	V1.0
SAP VERSION DATE	08/04/2023
PROTOCOL VERSION	V2
PROTOCOL VERSION DATE	26/04/2021
EUDRACT NUMBER	2020-000685-42
CLINICALTRIALS.GOV IDENTIFIER	NCT04553081
SPONSOR	Ghent University Hospital
TRIAL CHIEF INVESTIGATOR	Dr. Vandekerckhove Linos, MD, Department of Internal Medicine, Ghent University<br><br>Dr. De Scheerder Marie-Angélique, MD, Department of Internal Medicine, Ghent University
TRIAL STATISTICIAN	Dr. De Buyser Stefanie, Biostatistics Unit, Ghent University
SAP AUTHOR	Dr. De Buyser Stefanie, Biostatistics Unit, Ghent University
SAP REVIEWERS	Dr. Blomme Evy, Department of Internal Medicine, Ghent University<br><br>Dr. Degroote Sophie, Department of Internal Medicine, Ghent University<br><br>Dr. Jones Bryn, MD, Global medical lead, Dolutegavir, ViiV Healthcare, Brentford, UK<br><br>Dr. Marinus Wouter, MD, Belgian country medical director, ViiV Healthcare, Belgium<br><br>Dr. Liao Qiming, Senior Director, Statistics, ViiV Healthcare, Raleigh-Durham, NC, USA

SAP Revision History

Protocol version	Updated SAP version	Section changed	Description of and reason for change	Date changed	Timing
V2	V1.0	NA	NA	NA	Before study completion, after primary results have been presented by study researchers, prior to unblinding of the statistician

Table of Contents

List of abbreviations 5
Introduction 6
  Background and rationale 6
  Objectives 6
  Endpoints 6
Study Methods 9
  Trial design 9
  Interventions 9
  Randomization 9
  Sample size 9
  Statistical interim analyses and stopping guidance 10
  Timing of final analysis 10
  Timing of outcome assessments 10
Statistical Principles 10
  Confidence intervals and P values 10
  Adherence 11
  Analysis populations 11
Trial Population 11
  Screening data 11
  CONSORT Flow diagram 11
  Withdrawal / Follow-up 12
  Baseline patient characteristics 12
Analysis 13
  Outcome definitions 13
  Analysis methods 13
  Covariates 13
  Sensitivity analyses 14
  Subgroup analyses 14
  Missing data 14
  Additional analyses 14
  Safety analyses 15
  Statistical software 15
References 16

Signature page

Dr. De Buyser Stefanie (SAP author)<br>Stefanie De Buyser<br>Digitally signed by Stefanie De Buyser<br>Date: 2023.04.08 11:16:43 +02'00'
Dr. Vandekerckhove Linos (chief investigator)<br>Linos Vandekerckhove<br>Digitally signed by Linos Vandekerckhove<br>Date: 2023.04.11 11:01:49 +02'00'
Dr. Marie-Angélique De Scheerder (chief investigator)
Dr. Blomme Evy (SAP reviewer)<br>Evy Blomme<br>Digitally signed by Evy Blomme<br>Date: 2023.04.10 13:54:42 +02'00'
Dr. Degroote Sophie (SAP reviewer)<br>Sophie Degroote (Authentication)<br>Digitaal ondertekend door Sophie Degroote (Authentication)<br>Datum: 2023.04.11 11:15:33 +02'00'
Dr. Jones Bryn (SAP reviewer)
Dr. Marinus Wouter (SAP reviewer)
Dr. Liao Qiming (SAP reviewer)

List of abbreviations

2DR	Dual drug regimen
3DR	Triple drug regimen
ART	Antiretroviral therapy
D0	Day zero
D1	Day 1
FDA	Food and Drug Administration
HDL	High density lipoprotein
HIV	Human Immunodeficiency virus
HOMA-IR	Homeostatic model assessment for insulin resistance
IPDA	intact proviral DNA assay
ITT	Intention to treat
MICE	Multivariate Imputation by Chained Equations
SAP	Statistical analysis plan
TAF	Tenofovir alafenamide
W24	Week 24
W48	Week 48
W72	Week 72
W96	Week 96
W120	Week 120
W144	Week 144

Introduction

Background and rationale

Over the last years, there has been a paradigm change in HIV care for both naïve and switch patients, from triple therapy treatment to dual therapy treatment. Dual therapy comes with the advantages of lower cost, less side-effects, more preserved treatment options, and less interactions compared to triple therapy. However, comparative data on virologic and immunological parameters are lacking. In addition, further data is needed to investigate patterns of weight change and any associated metabolic impact caused by these newer antiretroviral drugs.

Objectives

Primary objective

The primary objective is to evaluate whether dual therapy with Dovato is non-inferior compared to triple therapy with Biktarvy, by a specified amount of 12% (called the non-inferiority margin),
with respect to the relative mean change from baseline at week 48 in number of intact replication competent HIV-1 DNA copies per million $CD4^+$ T cells (quantified by IPDA),
in adult HIV-1 infected patients with plasma HIV-1 RNA < 50 copies/mL who have been for at least 3 months on any stable 2nd generation integrase based triple therapy antiretroviral regimen before randomization.

After the last patient was randomized, we performed the power analysis for this endpoint (see the Section of Sample Size below for more details). Since the power is very low with the small sample size for the study, we consider the study is hypothesis-generating (not confirmatory) and no non-inferiority test will be conducted.

Secondary objectives

To compare dual therapy with Dovato versus triple therapy with Biktarvy with respect to

- metabolic health
- patient satisfaction
- virologic outcomes
- immune activation markers

in adult HIV-1 infected patients with plasma HIV-1 RNA < 50 copies/mL who have been for at least 3 months on any stable 2nd generation integrase based triple therapy antiretroviral regimen before randomization.

Endpoints

Primary efficacy endpoint

- Mean change from baseline at W48 in number of intact replication competent HIV-1 DNA copies per million $CD4^+$ T cells, quantified by the recently developed IPDA method (with DSI correction). Lower numbers are better.

Sensitivity endpoint related to the primary efficacy endpoint

- Mean change from baseline at W48 in number of intact HIV-1 DNA copies per million $CD4^+$ T cells (without DSI correction)

Key secondary efficacy endpoint

Virologic outcome

  - Mean change from baseline at W48 in ratio intact HIV-1 DNA copies / total HIV-1 DNA copies

Secondary efficacy endpoints

Metabolic health

- Mean change from baseline at W24, W48, W72, W96, W120 and W144 in **metabolic parameters**
  - Waist (cm)
  - Total cholesterol/HDL ratio
  - Leantrunk mass (g)
  - Fat percentage (%)
  - HOMA-IR
  - Fibro CAP (dB/m)

Exploratory endpoints

- Mean change from baseline at W24 in **patient satisfaction** [questionnaire with scores ranging between -3 and +3, scores > 0 indicate improvement]
- Mean change from baseline at W48 and W144 in **virologic outcomes**
  - Quantification of RNA transcripts
  - Total HIV-1 DNA copies per million $CD4^+$ T cells
  - Viral load (copies/ml)
- Mean change from baseline at W144 in virologic outcomes
  - Ratio intact HIV-1 DNA copies / total HIV-DNA copies
  - Intact cp per million $CD4^+$ T cells with DSI correction
  - Full length sequencing of the virus
- Mean change from baseline at W48 and W144 in **immunologic outcomes**
  - Inflammation markers
    - IL-6 (pg/ml)
    - CRP (mg/l)
    - IL-8 (pg/ml)
    - IP-10 (pg/ml)
    - B2M (pg/ml)
    - CXCL-1 (pg/ml)
    - IL-21 (pg/ml)
    - Hyaluronic acid (ng/ml)
  - Markers of coagulopathy
    - D-dimer (ng/ml)
  - Markers of microbial translocation
    - sCD14 (µg/ml)
  - CD4/CD8 ratio
  - IL-10 (pg/ml)
  - TNFa (pg/ml)
  - VCAM-1 (ng/ml)
  - sCD163 (ng/ml)
  - Immune cell subsets
    - Lymphocytes, Total T cells, helper T cells and subsets (naive, regulatory, central memory, transitional memory, (terminaly differentiated) effector

memory, early and early like effector, CD127-expressing, TSCM, TTM, TEMRA, and TTE)
    - cytotoxic T cells and subsets
    - γδ T cells and subsets (distinction based on CD4, CD8, CD25, CD27, CCR7, CD45RA)
    - CD4+CD8+ T cells, total B cells, mature naïve B cells, memory B cells, basophils, eosinophils, neutrophils, macrophages, immature granulocytes, normoblasts, NK cells and subsets (early, mature, terminal, NKG2A/C/D expressing), NKT cells and subsets, dendritic cells (classic and plasmacytoid DCs, CD80/CD86/CD33 expression), total monocytes, classical monocytes, non-classical monocytes, intermediate monocytes, CD33+CD14+ monocytes.
    - MFI and proportion of cell subsets expressing activation markers (CD25, CD38, HLA-DR, sCD27, sCD40), and exhaustion markers (PD-1, TIGIT, LAG-3, TIM-3, 2B4, CTLA4, CD28)
    - Cell proliferation(Ki67) and Terminal differentiation and proliferative history (CD57)
  - CD4 counts
  - CD8 counts
  - Function of immune cells
    - Cytotoxicity and cytokine secretion (NK and T cells)
    - Immune signaling (secretion of cytokines, chemokines, immune-stimulatory or immune-inhibitory factors upon in vitro cell stimulation)
- Mean change from baseline at W48 and W144 in **metabolic outcomes**
  - Waist (cm)
  - BMI (kg/m²)
  - ALT (U/l)
  - AST (U/l)
  - AST/ALT ratio
  - FIB4
  - Insulin (mU/l)
  - Glucose (mg/dl)
  - Triglycerids (mg/dl)
  - Cholesterol (mg/dl)
  - LDL (mg/dl)
  - HDL (mg/dl)
  - HBa1c (%)
  - HBa1c mol (mmol/mol)
  - Leantrunk (g)
  - Leanlimb (g)
  - Trunkfat (g)
  - Limbfat (g)
  - Fibro lsm (kPa)
  - Total fat mass (g)
  - Metabolic syndrome (incidence)
  - Appendiculair lean/height² (kg/m²)
  - Est. VAT (estimated visceral adipose tissue) mass (g)
  - Android/gynoid ratio
  - Total lean body mass (g)

  - Blood pressure

Safety endpoints

- Adverse events and subclassifications thereof
  - AEs leading to death
  - AEs leading to discontinuation of study treatment
  - Suspected unexpected serious adverse reactions (SUSARs)
  - Serious adverse reactions (SARs)
  - Adverse reactions (ARs)
  - Serious adverse events (SAEs)
  - AEs not including SAEs that exceed a frequency threshold of 5%

Study Methods

Trial design

- Randomized controlled
- Parallel group design
- Two-arm
- Open-label
- Monocentric
- Phase 4

Interventions

Active control = Triple therapy (3DR)

Biktarvy (bictegravir 50mg/emtricitabine 200mg/tenofovir alafenamide 25mg) (BIC/TAF/FTC): one tablet a day

Test drug = Dual therapy (2DR)

Dovato (DTG 50mg/lamivudine 300mg): one tablet a day

Randomization

- Block randomization with fixed block size
- Patients will be randomized to switch to / stay on the triple regimen or to switch to the dual regimen
- 2:1 allocation ratio (2DR:3DR)
- Randomization was not stratified

Sample size

- 134 subjects

The sample size calculation mentioned in the protocol was aimed to power the analysis for the key secondary endpoint (ratio intact HIV-1 DNA copies / total HIV-DNA copies), because the primary endpoint is based on the recently developed IPDA method with limited literature data. Unfortunately, it mistakenly assumed that the key secondary endpoint was binary.

After the last patient was randomized, power calculations were performed with respect to the primary endpoint, based on the publications of Bruner et al. (2019) and Dragoni et al. (2022).

Based on figure 3.f in the paper from Bruner et al. (2019), the geometric mean was estimated to be 105 intact replication competent HIV-1 sequences per million $CD4^+$ T cells, and the SD of the log-transformed rate was estimated at 1.54. A sample size of 134 patients in total (89 on 2DR:45 on 3DR) would only achieve 7.5% power to conclude non-inferiority of 2DR compared to 3DR at the 2.5% one-sided significance level with respect to mean number of intact replication competent HIV-1 copies per million $CD4^+$ T cells at W48, assuming both regimens are equally effective (true mean difference between groups = 0%), the standard deviation of the log-transformed rate is 1.54 in both groups, and the compliance rate is 90%, when the non-inferiority margin for the mean relative difference is set at 12% of the expected geometric mean with 3DR.

In the paper from Dragoni et al. (2022), the interquartile range for the change from baseline at W48 in $\log_{10}$ intact HIV-1 DNA copies per million $CD4^+$ T cells in patients on 3DRs goes from -0.43 to -0.01. Assuming a lognormal distribution, the standard deviation for the change was estimated at 0.31 ($\log_{10}$ scale). A sample size of 134 patients in total (89 on 2DR:45 on 3DR) would only achieve 13% power to conclude non-inferiority of 2DR compared to 3DR at the 2.5% one-sided significance level with respect to change from baseline in mean number of intact replication competent HIV-1 copies per million $CD4^+$ T cells at W48, assuming both regimens are equally effective (true mean difference between groups = 0%), the standard deviation of the difference between $\log_{10}$-transformed rates is 0.31 in both groups, and the compliance rate is 90%, when the non-inferiority margin for the mean relative difference is set at 12% of the expected geometric mean with 3DR.

Power calculations were performed using the tTestPower() function from the EnvStats package in R. The main calculation was verified using validated SAS software (proc power).

Statistical interim analyses and stopping guidance

No interim analysis for efficacy nor for futility has been foreseen.

Timing of final analysis

- Endpoints are analyzed only after all patients have complete follow-up on the respective endpoint
- Endpoints are not analyzed collectively but when available (depending on length of follow-up required)

Timing of outcome assessments

Data are collected at screening (D0), D1, W24, W48, W72, W96, W120, and W144 (with visit windows of 4 weeks).

The number of intact replication competent HIV-1 sequences per million $CD4^+$ T cells is quantified by IPDA on D1, W48 and W144. The primary endpoint is relative change from D1 at W48 (W48/D1).

Metabolic parameters are measured on D1, W24, W48, W72, W96, W120 and W144.

Virologic and immunologic endpoints are measured on D1, W48 and W144 (except for full-length and integration site sequencing of the virus, which will be performed on sorted samples at D1 and W144).

Statistical Principles

Confidence intervals and P values

All reported confidence intervals (CIs) for estimates of between-group effects will be two-sided 95% CIs. No p-values will be reported, analyses should be considered as hypothesis-generating (not confirmatory) and interpreted with care

Adherence

Adherence will be assessed using a surrogate marker, plasma viral load. Nonadherence is defined as either a recurrent viral load above 50 copies/ml or in case of a single viral load, a measurement above 200 copies/ml.

Analysis populations

ITT-E

The analysis on the intention-to-treat (ITT) population will include all randomized patients who have received at least one dose of study treatment and analyze them according to the allocated intervention arm, irrespective of patients' compliance with the planned intervention.

Per protocol

A per protocol analysis will be performed if more than 5% of 2DR patients either switched to 3DR or were nonadherent. The per protocol population will include all randomized patients who complied with the allocated treatment sufficiently to ensure that these data would be likely to exhibit the effects of treatment, according to the underlying scientific model. Patients allocated to 2DR, who switch to or stay on 3DR will be excluded from the per protocol analysis. In case of a recurrent viral load above 50 copies/ml or in case of a single viral load measurement above 200 copies/ml, the patient will also be excluded from the per protocol population.

Trial Population

Screening data

The total number of adult HIV-1 infected patients who were assessed for eligibility was not be collected during the conduct of this study as it was considered heavy on resources and it would not be sufficiently reliable.

CONSORT Flow diagram

The flow of participants through the trial will be summarized using a CONSORT flow diagram. The flow diagram will summarize the number of patients who gave consent to participate and who

- gave informed consent
- were randomized
- were randomized but not eligible
- were allocated to each intervention (2DR or 3DR)
- received the allocated intervention
    - received allocated intervention and were fully compliant
    - received allocated intervention, but were not fully compliant
- did not receive allocated intervention
    - withdrew consent before receiving allocated intervention
    - other reason why the allocated intervention was not received
- completed the study until W48
- did not complete the study until W48
    - withdrew consent
    - died

- lost to follow-up
- were included in the primary analysis on the primary endpoint
- completed the study until follow-up at W144
- did not complete the study until follow-up at W144
  - withdrew consent
  - died
  - were lost to follow-up

Withdrawal / Follow-up

The numbers of losses to follow-up (drop-outs, withdrawals from allocated treatment and withdrawals from study) will be summarized by treatment arm.

Baseline patient characteristics

The following baseline characteristics will be summarized:

- Smoking status
- Drug use (elicit or recreational)
- Sexual orientation
- Ethnicity
- HIV history
  - Time since diagnosis
  - Years on ART
  - HIV subtype
  - ART history
  - ART Regimen at Screening
- Concomitant medication: focus on metabolic agents (antidiabetics, lipid lowering agents, anti-hypertensive medication, antiplatelet agents, neuropsychiatric medication)
- Sex (M/F)
- Age (year)
- CD4 at screening (cells/µl)
- CD4 nadir (cells/µl)
- Peak viral load (copies/ml plasma)
- Time on ART (year)
- Time from start ART to undetectable viral load (year)
- Time since diagnosis to start ART (year)
- Total HIV-1 DNA copies/10^6 $CD4^+$ T cells
- Intact proviral HIV-1 DNA copies/10^6 $CD4^+$ T cells
- Waist (cm)
- BMI (kg/m^2)
- ALT (U/l)
- AST (U/l)
- AST/ALT ratio
- FIB4
- Insulin (mU/l)
- Glucose (mg/dl)
- Triglycerids (mg/dl)
- Cholesterol (mg/dl)
- LDL (mg/dl)
- HDL (mg/dl)
- HBa1c (%)

Normally distributed data is summarized using the arithmetic mean and standard deviation. Lognormally distributed data is summarized using the geometric mean and geometric coefficient of variation. Not (log)normally distributed numeric data is summarized using the median with 25th and 75th percentiles. Categorical variables are presented by absolute and relative frequencies.

Analysis

Outcome definitions

Lab assessments which fall below the detection limit, will be given the value half of the detection limit.

Changes from baseline are computed as the absolute differences of post minus baseline. In case variables are lognormally distributed, the absolute difference of the $\log_{10}$-transformed variables is taken, which corresponds to the logarithm of the relative differences of the untransformed variables. Baseline measurements were scheduled at day of randomization (visit window of 4 weeks) or the day before at screening (if screening and randomization could not be planned at the same day, because of required additional blood analyses to check the inclusion and exclusion criteria).

Analysis methods

Ordinary linear regression models will be fitted for the change from baseline measurements. The models will include group, the baseline response value, and other baseline covariates as specified in the section on covariates below.

For normally distributed variables, the estimated arithmetic mean difference in change from baseline between groups will be reported with 95% Wald CI and p-value.

For lognormally distributed variables, the model fitting is based on log-transformed data and results are back-transformed to the original scale. The estimated geometric mean ratio of the groups' (2DR/3DR) relative change from baseline (W48/D1) is reported with 95% Wald CI and p-value.

For the primary and secondary endpoints, the estimated marginal mean responses will plotted by group together with individual patient data.

Covariates

The FDA recommends to adjust for baseline covariates that are anticipated to be most strongly associated with the outcome of interest. Adjustment for these baseline covariates will generally reduce the variability of estimation of treatment effects and thus lead to narrower confidence intervals and more powerful hypothesis testing. All analyses will be adjusted for the respective baseline response value. In addition:

- Analyses of the virologic endpoints will be adjusted for baseline CD4 nadir and time on ART (before randomization)
  - Analyses of the primary endpoint will be additionally adjusted for peak viral load and baseline regimen, but only if adjustment for these variables improves the model fit (evaluated through Akaike's Information Criterion)
- Analyses of the metabolic endpoints will be adjusted for BMI category (< 25 kg/m², [25-30] kg/m², > 30 kg/m²) and baseline regimen
- Analyses on the immunologic endpoints will be adjusted for age category (≤ 50 y, > 50 y), smoking status (current smoker, ex-smoker, never smoker), and baseline CD4/CD8 ratio

Sensitivity analyses

Analyses of the primary endpoint will additionally be adjusted for baseline total HIV-1 DNA copies per million $CD4^+$ T cells

Subgroup analyses

Subgroup analyses will be performed to explore the uniformity of the treatment effect on the primary and secondary endpoints.

Subgroups will be made according to

- TAF group (No TAF, stop TAF, start TAF, stay on TAF)
- Baseline regimen (Triumeq, Biktarvy, DTG/TDF with TAF, DTG/TAF with FTC, other)
- Age category (≤ 50 y, > 50 y)
- BMI category (< 25 kg/m², [25-30] kg/m², > 30 kg/m²)
- Gender (male, female)
- Ethnicity (European, African, Other)
- Comedication for hypertension at baseline
- Comedication for lipids at baseline
- Comedication for diabetics at baseline
- Comedication neuropsychiatric at baseline

Missing data

We assume missing data to be missing at random (MAR), which we will handle with multivariate imputations by chained equations (MICE). The imputation model will be specified separately for each variable. Imputations of numeric variables will be generated according to predictive mean matching. All variables that appear in the analysis model(s) for the missing endpoint will be included as predictors into the imputation model. The visiting scheme will order these variables according to their number of missing cases. The number of iterations was set at 20 to stabilize the distribution of the parameters. The number of multiply imputed data sets was set at 50 to minimize simulation error. The 50 imputed sets will be identical for the non-missing data entries, but different in the imputed values. The magnitude of these difference reflects our uncertainty about what value to impute. Differences between regression estimates from imputed datasets are caused because of our uncertainty about what value to impute. The regression estimates from each imputed dataset are pooled into one estimate. The R package mice will be used.

Withdrawal

Data collected after day of withdrawal will not be used for analysis.

Additional analyses

Objectives of additional analyses are:

- To evaluate the association of mean changes from baseline at W48 or W144 in virologic outcomes with mean changes from baseline at W48 or W144 in immunologic outcomes
- To evaluate the association of mean changes from baseline at W48 or W144 in immunologic outcomes with mean changes from baseline at W48 or W144 in metabolic outcomes

in adult HIV-1 infected patients with plasma HIV-1 RNA < 50 copies/mL who have been for at least 3 months on any stable 2nd generation integrase based triple therapy antiretroviral regimen before randomization.

To this end, we will present matrices of the estimated Pearson correlation coefficients ρ with p-value. Strong correlations ($\rho \geq 0.5$) which are statistically significant will be further analyzed in a linear regression model.

As these additional analyses don't make use of the randomized controlled design, no causal interpretations should be given to the associations.

Safety analyses

All safety variables will be summarized by actual treatment group using descriptive statistics. Statistical tests to compare differences between treatment groups regarding safety variables are not pre-specified.

Adverse events

Considered sub-classifications of AEs are: AEs leading to death, AEs leading to discontinuation of study intervention, SUSAEs, SAEs, and AEs not including SAEs that exceed a frequency threshold of 5%.

Tables of absolute and relative frequencies will be provided to summarize according to actual treatment group:

- The total number of AEs or any sub-classification thereof. Subjects with multiple AEs are counted more than once.
- The overall number of subjects having at least one AE or any sub-classification thereof. Each subject will only be counted once and any repetition will be ignored; the denominator will be the total population size.
- The number of subjects having at least one AE or any sub-classification thereof by body or organ system and preferred term drawn from the Common Terminology Criteria for Adverse Events (CTCAE) version 5.0. Subjects with multiple AEs with different preferred terms are counted more than once.

Listings by patient will be provided for: all AEs leading to death, all AEs leading to discontinuation of study treatment, and all SAEs. All of the above events (including the same event on several occasions) are listed for each patient together with the patient identifier, age, sex, actual treatment received, and the collected information regarding the AE.

Statistical software

Analyses will be performed using R version 4.2.2. Figures will be made using the base plotting system. Multiple imputations will be performed using the mice package.

References

Bruner KM, Wang Z, Simonetti FR, et al. (2019) A quantitative approach for measuring the reservoir of latent HIV-1 proviruses. Nature

Dragoni F, Rossetti B, Lombardi F, et al. (2022) Dynamics of Total and Intact HIV-1 DNA in Virologically Suppressed Patients Switching to DTG-Based or ATV-Based Dual Therapy. J Acquir Immune Defic Syndr

FDA Guidance (November 2016) Non-Inferiority Clinical Trials to Establish Effectiveness

FDA Guidance (January 2017) Multiple Endpoints in Clinical Trials

FDA Guidance (May 2021) Adjusting for Covariates in Randomized Clinical Trials for Drugs and Biological Products

Gamble C, Krishan A, Stocken D, et al. (2017) Guidelines for the content of statistical analysis plans in clinical trials. JAMA

R Core Team (2020). R: A language and environment for statistical computing. R Foundation for Statistical Computing, Vienna, Austria.

Van Buuren S and Groothuis-Oudshoorn K (2011). Mice: Multivariate Imputation by Chained Equations in R. Journal of Statistical Software
