# Supplementary material for: In-depth Analysis of the HIV Reservoir Confirms Effectiveness and Safety of Dolutegravir/Lamivudine in a Phase 4 Randomized Controlled Switch Trial (RUMBA)
Source: J Infect Dis. 2024 Sep 3;231(1):e91–e100. doi: 10.1093/infdis/jiae405 (PMC11793038; doi:10.1093/infdis/jiae405)
Supplement: jiae405_Supplementary_Data [file jiae405_supplementary_data.zip › Supplementary_material_Rumba_study (2).docx]

# Supplementary file 1

**Inclusion and exclusion criteria**

| **Inclusion criteria** |
| --- |
| - Age ≥18 years |
| - Ability and willingness to provide written informed consent. |
| - Ability to attend the complete schedule of assessments and patient visits. |
| - Ability and willingness to have blood samples collected and stored indefinitely and used for various research purposes. |
| - HIV RNA < 50 copies/mL for at least 3 months on a 2^nd^ generation integrase inhibitor (INSTI) based regimen |
| - Females of childbearing potential should be on effective contraception |
| **Exclusion criteria** |
| - Current presence of opportunistic infection (AIDS defining events as defined in category C of the CDC clinical classification). |
| - Evidence of active HBV infection (Hepatitis B surface antigen positive or HBV viral load positive in the past and no evidence of subsequent seroconversion (seroconversion= HBV antigen or viral load negative and positive HBV surface antibody). |
| - Evidence of active HCV infection: HCV antibody positive result within 60 days prior to study entry with positive HCV viral load or, if the HCV antibody result is negative, a positive HCV RNA result within 60 days prior to study entry. |
| - Pregnancy or breastfeeding. |
| - Patients unable to understand the study protocol or any other condition that in the investigator’s opinion may compromise compliance with the study protocol |
| - Decompensated liver cirrhosis (Child-Pugh B/C) - Unstable liver disease (as defined by the presence of ascites, encephalopathy, coagulopathy, hypoalbuminemia, oesophageal or gastric varices, or persistent jaundice), cirrhosis, known biliary abnormalities (apart from hyperbilirubinemia or jaundice due to Gilbert's syndrome or asymptomatic gallstones) |
| - Psychiatric and psychological disorders, which in the opinion of the investigator, will interfere with the trial conduct or safety of the participant. |
| - Previous participation in a trial evaluating an immune modulating agent. |
| - Active drug or alcohol use/addiction such that, in the opinion of the site investigator, would interfere with adherence to study requirements. |
| - Treatment failure on an integrase inhibitor containing regimen and reported baseline resistance |
| - Creatinine Clearance <50 |
| - Tuberculosis treatment |
| - Documented M184V |
| - Previous virological failure >200 copies/mL on NRTI |
| - Subjects with history or presence of allergy to any of the study drugs or their components |
| - ALT >5 times the ULN, OR ALT >3xULN and bilirubin >1.5xULN (with >35% direct bilirubin) |

**Table S1: Overview of all data collected and detailed methods on collection and analyses**

| Endpoints | Source |
| --- | --- |
| **Primary efficacy endpoint** | |
| Mean change from baseline at W48 in number of intact HIV-1 DNA copies per million CD4+ T cells (with DNA Shearing Index (DSI) correction) | EasySep Human CD4+ T cell isolation kit: STEMCELL Technologies |
|  | DNeasy Blood & Tissue kit (Qiagen) |
|  | Qubit dsDNA BR Assay Kit (Invitrogen, Thermo Fisher Scientific) |
|  | Qubit 30 fluorometer (Invitrogen, Life technologies). |
|  | Primer and probe sequences for the Cross-Subtype IPDA were acquired from Cassidy et al. [1]and synthesised by Integrated DNA Technologies (IDT). |
|  | *RPP30 assay* according to Bruner et al.[2] |
|  | 26K 24-well nanoplate (ID: 250001, Qiagen) |
|  | QIAcuity probe mix (Qiagen) |
|  | QIAcuity Four system (Qiagen) |
| **Sensitivity endpoint related to the primary efficacy endpoint** | |
| Mean change from baseline at W48 in number of intact HIV-1 DNA copies per million CD4+ T cells (without DSI correction) |  |
| **Secondary efficacy endpoints** | |
| Mean change from baseline at W24, W48, W72, W96, W120 and W144 in metabolic parameters | |
| o Weight (kg) | Standardized clinical exam: calibrated scale |
| o Total cholesterol/HDL ratio | Abott Architect C device |
| o Lean trunk mass (g) | DEXA scans: Hologic QDR Horizon A device (series number 301096M, software version Apex v5.6.0.5), total body scans were done in Array scan modus. |
| o Fat percentage (%) |  |
| o HOMA-IR | Cobas e801 Roche device |
| o Fibro CAP (dB/m) | Fibroscan 430+ Mini (SN F92775) (M probe SN 0078874 and XL probe SN 0095376, software release number FS 4.1.3) and Fibroscan 530 Compact (SN F80175) (M probe, software G3.2) |
| **Exploratory endpoints** |  |
| **Mean change from baseline at W48 and W144 in virologic outcomes** | |
| o Quantification of RNA transcripts | Primer and probe sequences for RNA transcripts were acquired from Yukl et al. [3] |
|  | RNeasy Plus Mini Kit (Qiagen) |
|  | QIAcube Connect (Qiagen) |
|  | Qubit RNA BR Assay Kit (Invitrogen, Thermo Fisher Scientific) |
| TAR assay | cDNA: Poly(A) Polymerase Tailing Kit (Biosearch technologies) |
| Yukl assays | cDNA: SuperScript III First-Strand kit (Invitrogen, Thermo Fisher Scientific) |
|  | T100 Thermal Cycler (Bio-Rad) |
|  | SimpliAmp Thermal Cycler (Applied biosystems, Thermo Fisher Scientific) |
|  | 26K 24-well nanoplate (ID: 250001, Qiagen) |
|  | QIAcuity probe mix (Qiagen) |
|  | QIAcuity Four system (Qiagen) |
|  | Reference genes (*TBP*, *YWHAZ*, *B2M* and *HPRT*) were measured with the LightCycler 480 II |
|  | LightCycler 480 SYBR Green I Master mix (Roche).  The TAR assay and an in-house multiplex Rainbow transcriptional RNA dPCR assay for long LTR, pol and tat-rev were run in duplicate on participants with subtype B on the QIAcuity Four system (Qiagen). The digital PCR program started with 2 minutes at 95°C, followed by 45 cycles of 94°C for 30 seconds and 59°C for 60 seconds. RNA concentrations and reference genes were used to normalize HIV-1 RNA transcripts [4]. Data analysis was performed with an in-house R script, a modified version of ddpcrquant to accommodate QIAcuity data analysis. |
| o Total HIV-1 DNA copies per million CD4+ T cells |  |
| o Plasma viral load (copies/ml) | Cobas AmpliPrep-Cobas TaqMan HIV-1 test, version 2.0; Roche Diagnostic, detection limit 20 copies/ml |
| **Mean change from baseline at W48 and W144 in immunologic outcomes** | |
|  | Fresh plasma was collected within 1 hour after venipuncture, aliquoted and stored at -80ºC until analysis. Samples were assayed in order of screening number (randomized for treatment) in batches containing all samples from the same individual. Samples were pre-diluted 1:200 1:1000 and 1:2000 for sCD163, VCAM-1 and sCD14 respectively. Measurements below the detection limit were replaced by a half detection limit. Multiskan FC Microplate Photometer (Thermo Scientific) and Luminex 100/200 system (Luminex) |
| o Inflammation markers |  |
| ▪ IL-6 (pg/ml) | Quantikine HS Human IL-6, R&D Systems, USA) |
| ▪ CRP (mg/l) | photometric measurement, Architect c16000, Abbott, Abbott Laboratories, Illinois, United States |
| ▪ IL-8 (pg/ml) | R&D Luminex HS assay (R&D Systems, USA)) |
| ▪ IP-10 (pg/ml) | R&D Luminex Discovery assay (R&D Systems, USA) |
| ▪ CXCL-1 (pg/ml) | Duoset ELISA kits, R&D systems, USA |
| ▪ IL-21 (pg/ml) | Duoset ELISA kits, R&D systems, USA |
| o Markers of coagulopathy |  |
| ▪ D-dimer (ng/ml) | immunoturbidimetry, STA R Max2, Stago, France |
| o Markers of microbial translocation |  |
| ▪ sCD14 (µg/ml) | Duoset ELISA kits, R&D systems, USA |
| o CD4/CD8 ratio | Sysmex XN-1000 |
| o IL-10 (pg/ml) | R&D Luminex HS assay (R&D Systems, USA)) |
| o TNFα (pg/ml) | Quantikine HS Human TNF-α, R&D Systems, USA) |
| o VCAM-1 (ng/ml) | Duoset ELISA kits, R&D systems, USA |
| o sCD163 (ng/ml) | Duoset ELISA kits, R&D systems, USA |
| o Immune cell subsets | PBMCs in FCS/DMSO 10% were stored in liquid nitrogen after Ficoll gradient centrifugation. On the day of the assay, PBMCs from both timepoints were thawed according to the protocol of Barcelo et al. [5] Cells were pre-blocked with 5% FcX Trustain Fc block (Biolegend) for 10 min. After washing, cells were stained for 20 min at room temperature with different monoclonal antibodies in Brilliant Stain Buffer PLUS (BD) containing Fixable Viability Dye 780. Samples were fixed with PFA and on average >1 million cells were acquired the next day on a BD FACSymphony A3 instrument (BD Biosciences, Spain) and analysed using FlowJo v10.8.1 software. Via the PeacoQC algorithm [6] good quality events were selected for further downstream analysis. |
| ▪ Lymphocytes, T cells and subsets (based on CD45RO, CCR7 and CD27 expression), CD4+CD8+ T cells, naïve, class and non-class switched memory B cells and late memory B cells, immature granulocytes, normoblasts, NK cells and subsets (early, mature, terminal, NKG2A expressing), NKT cells and subsets, dendritic cells (classic and plasmacytoid DCs, CD80/CD86/CD33 expression), total monocytes, classical monocytes, nonclassical monocytes, intermediate monocytes. | Panel 1: CD4 BV750, CD33 PE-Cy5, CD159A (NKG2A), APC CD123 PE, CD16 PE-Cy7, CD14 BV570, CD127 BV605 (Biolegend), CD56 PerCP-Cy5.5, CD3 PE/Dazzle™ 594, CD80 BV650, CD11c FITC, HLA-DR BV786, CD8 V500, CD86 BV711 (BD Biosciences). Panel 2: CD45RA PE-Cy7, CD19 Alexa Fluor 700, CD4 PerCP-Cy5.5, PD-1 PE, CD45RO BV570, CD3 FITC, TCR γ/δ PE/Dazzle™ 594 (Biolegend), CD27 BV750, IgD BV711, CD38 BV605, CCR7 BV421, CD25 BV650 (BD Biosciences), TIGIT APC, LAG-3 PE-Cy5 (Thermofisher). Positive gates were determined based on ‘fluorescence minus one’ controls. |
|  |  |
|  |  |
| ▪ MFI and proportion of cell subsets expressing activation markers (CD25, CD38, HLA-DR), and exhaustion markers (PD-1, TIGIT, LAG-3) |  |
| **Mean change from baseline at W48 and W144 in metabolic outcomes** | |
| o Waist (cm) | Standardized clinical exam: a ribbon meter at the umbilicus. |
| o BMI (kg/m²) | Standardized clinical exam: calibrated devices |
| o Blood pressure |  |
| o ALT (U/l) | Abott Architect C device |
| o AST (U/l) |  |
| o AST/ALT ratio |  |
| o Fibro lsm (kPa) | Fibroscans were executed with Fibroscan 430+ Mini (SN F92775) (M probe SN 0078874 and XL probe SN 0095376, software release number FS 4.1.3) and Fibroscan 530 Compact (SN F80175) (M probe, software G3.2). |
| o Insulin (mU/l) | Cobas e801 Roche device |
| o Glucose (mg/dl) | Abott Architect C device |
| o Triglycerids (mg/dl) |  |
| o Cholesterol (mg/dl) |  |
| o LDL (mg/dl) |  |
| o HDL (mg/dl) |  |
| o HBa1c (%) | Tosoh HLV-723 G8 device |
| o HBa1c mol (mmol/mol) |  |
| o Lean trunk (g) | DEXA scans: Hologic QDR Horizon A device (series number 301096M, software version Apex v5.6.0.5), total body scans were done in Array scan modus. |
| o Lean limb (g) |  |
| o Trunk fat (g) |  |
| o Limb fat (g) |  |
| o Total fat mass (g) |  |
| o Appendicular lean/height² (kg/m²) |  |
| o Est. VAT (estimated visceral adipose tissue) mass (g) |  |
| o Android/gynoid ratio |  |
| o Total lean body mass (g) |  |

**Table S2**

| CS-IPDA | Sequence | Reference |
| --- | --- | --- |
| TOTAL Fwd primer | TTAAGCCTCAATAAAGCTTGCC | [7] |
| TOTAL Rvd primer | GTTCGGGCGCCACTGCTAGA |  |
| TOTAL probe | /56ROXN/CCAGAGTCACACAACAGACGGGCACA/3IAbRQSp/ | [8] |
| LTR-GAG Fwd primer | GACTAGCGGAGGCTAGAAGGAGAGA | [1] |
| LTR-GAG Rvd primer | CTAATTTTCCSCCDCTTAATAYTGACG |  |
| LTR-GAG probe | /5Cy5/AT+G+GGT+GC+GAGA/3IAbRQSp/ |  |
| ENV Fwd primer | TVTTCMTTGGGTTCTTRGGAGCAGCAGG |  |
| ENV Rvd primer | GCACTATRCCAGACAATAVYTGTCTGGCCTGTACC |  |
| ENV probe | /5HEX/A+G+CA+CKA+T+G+GG/3IABkFQ/ |  |
| 5’ POL Fwd primer | WCCYTTARYTTCCCTCARATCACTCT |  |
| 5’ POL Rvd primer | TACTGTATCATCTGCTCCTGTRTCTAAKAGAGCYTC |  |
| 5’ POL probe | /56-FAM/TTG+G+CARC+GA+CC/3IABkFQ/ |  |

**Table S3**

| CS-IPDA + RU5 |  |  |  |
| --- | --- | --- | --- |
|  | conc (µM) | wanted (µM) | for 1 (µl) |
| 4x probe mix |  |  | 10 |
| CS-ENV | 16.6/16.6/5 | 0.789/0.789/0.238 | 1,9 |
| 5'Pol | 16.6/16.6/5 | 0.913/0.913/0.275 | 2,2 |
| LTR-GAG | 16.6/16.6/5 | 0.789/0.789/0.238 | 1,9 |
| RU5 ROX | 20x | 0.900/0.900/0.250 | 2 |
| xbai | 100000 units/ml |  | 0,3 |
| water |  |  | 15,7 |
| target |  |  | 6 |
| total |  |  | 40 |

**Figure S1: Consort diagram**


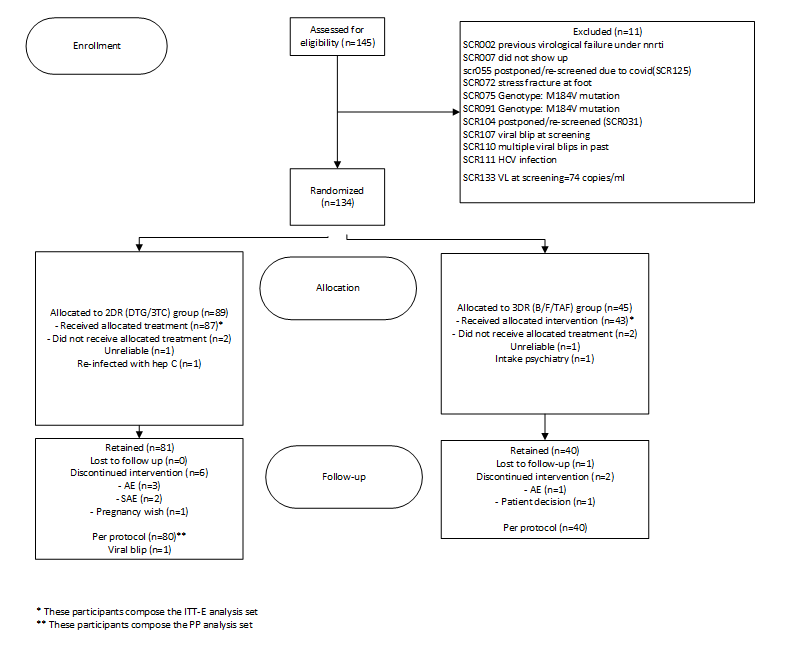


**Table S4: Adverse events leading to discontinuation of study treatment and SAEs**

| Number | Age | Sex | Treatment arm | AE | Outcome |
| --- | --- | --- | --- | --- | --- |
| SCR083 | 47y | male | DTG/3TC | diarrhea & vivid dreams | discontinuation of study treatment |
| SCR144 | 37y | male | DTG/3TC | excessive dreaming & feeling down | discontinuation of study treatment |
| SCR020 | 31y | male | B/F/TAF | diarrhea | discontinuation of study treatment |
| SCR070 | 56y | male | DTG/3TC | headache & fatigue & stomach pain | discontinuation of study treatment |
| SCR093 | 40y | male | DTG/3TC | acute psychosis | SAE: hospitalization |

**Table S5: soluble markers and immune cell relative frequencies**

|  | **ITT-E** | | | **B/F/TAF (3DR)** | | | **DTG/3TC (2DR)** | | |
| --- | --- | --- | --- | --- | --- | --- | --- | --- | --- |
|  | **N=130** | | | **N=43** | | | **N=87** | | |
|  | **Treatment ratio** | | | **W48 visit to baseline ratio** | | | **W48 visit to baseline ratio** | | |
|  | **Estimate** | **95% LCL** | **95% UCL** | **Estimate** | **95% LCL** | **95% UCL** | **Estimate** | **95% LCL** | **95% UCL** |
| **IL-6** | 0,98 | 0,75 | 1,28 | 0,96 | 0,75 | 1,22 | 0,98 | 0,84 | 1,14 |
| **sCD14** | 0,99 | 0,92 | 1,06 | 1,01 | 0,95 | 1,08 | 1,02 | 0,98 | 1,07 |
| **sCD163** | 1,05 | 0,93 | 1,18 | 1,09 | 0,96 | 1,23 | 1,04 | 0,96 | 1,13 |
| **VCAM-1** | 0,98 | 0,91 | 1,06 | 0,97 | 0,90 | 1,04 | 0,98 | 0,94 | 1,04 |
| **TNF-α** | 1,02 | 0,91 | 1,14 | 1,04 | 0,94 | 1,16 | 1,02 | 0,95 | 1,10 |
| **IP-10** | 1,11 | 0,95 | 1,29 | 1,10 | 0,95 | 1,27 | 0,99 | 0,90 | 1,10 |
| **IL-8** | 0,88 | 0,64 | 1,20 | 0,96 | 0,71 | 1,30 | 1,10 | 0,90 | 1,35 |
| **D-dimer** | 1,17 | 0,91 | 1,50 | 1,17 | 0,94 | 1,46 | 1,00 | 0,86 | 1,15 |
| **Lymphocytes** | 0,98 | 0,90 | 1,06 | 0,93 | 0,87 | 1,00 | 0,95 | 0,91 | 1,00 |
| **CD4** | 0,94 | 0,82 | 1,07 | 0,93 | 0,82 | 1,04 | 0,99 | 0,91 | 1,07 |
| **CD8** | 0,95 | 0,84 | 1,08 | 0,93 | 0,83 | 1,04 | 0,97 | 0,91 | 1,05 |
| **CD4/CD8 ratio** | 1,01 | 0,88 | 1,15 | 1,10 | 0,98 | 1,25 | 1,10 | 1,01 | 1,20 |
| **NK T** | 1,03 | 0,83 | 1,27 | 0,98 | 0,80 | 1,20 | 0,95 | 0,82 | 1,10 |
| **γδ T** | 0,90 | ***0,81*** | ***0,99*** | 0,91 | 0,83 | 1,00 | 1,02 | 0,95 | 1,09 |
| **B cells** | 0,90 | 0,80 | 1,00 | 1,00 | 0,89 | 1,13 | 1,11 | 1,03 | 1,21 |
| **NK** | 1,05 | 0,91 | 1,22 | 1,10 | 0,96 | 1,27 | 1,05 | 0,95 | 1,16 |
| **Monocytes** | 0,95 | 0,85 | 1,07 | 1,02 | 0,92 | 1,13 | 1,07 | 1,00 | 1,15 |
| **DC** | 1,00 | 0,97 | 1,02 | 0,99 | 0,97 | 1,01 | 0,99 | 0,98 | 1,01 |

Immunological changes after 48 weeks, corrected for age category (≤ 50 y, > 50 y), CD4/CD8 ratio, smoking status and baseline response value in the ITT-E population. The confidence intervals for the treatment ratios in bold and italic do not contain the null value. No statistic models were fitted on CRP, IL-10, IL-21, and CXCL-1, because more than 75% of the measurements were below the limit of detection. Nor were models fitted on the number of immature granulophils and normoblasts, because there were less than 20 unique observations.

**Table S6: Secondary and exploratory metabolic outcomes**

|  |  |  | **ITT-E** | | | **B/F/TAF (3DR)** | | | **3TC/DTG (2DR)** | | |
| --- | --- | --- | --- | --- | --- | --- | --- | --- | --- | --- | --- |
|  |  |  |  | N=130 |  | N=43 | | | N=87 | | |
|  |  |  | Treatment ratio 3DR/2DR | | | W48 visit to baseline ratio | | | W48 visit to baseline ratio | | |
| **Endpoint** | **Population** | **N** | **Estimate** | **95% LCL** | **95% UCL** | **Estimate** | **95% LCL** | **95% UCL** | **Estimate** | **95% LCL** | **95% UCL** |
| **Weight** | ITT-E | 130 | 0,99 | 0,97 | 1,02 | 0,97 | 0,93 | 1,02 | 0,98 | 0,94 | 1,02 |
| **Weight** | PP | 120 | 0,99 | 0,97 | 1,02 | 0,98 | 0,94 | 1,02 | 0,98 | 0,94 | 1,02 |
| **Cholesterol/HDL ratio** | ITT-E | 130 | 1,02 | 0,94 | 1,11 | 0,96 | 0,83 | 1,11 | 0,94 | 0,82 | 1,08 |
| **Cholesterol/HDL ratio** | PP | 120 | 1,03 | 0,95 | 1,12 | 0,97 | 0,84 | 1,12 | 0,94 | 0,83 | 1,08 |
| **Trunk lean mass** | ITT-E | 130 | 0,97 | 0,95 | 1,00 | 0,96 | 0,93 | 1,00 | 0,99 | 0,96 | 1,03 |
| **Trunk lean mass** | PP | 120 | 0,97 | 0,95 | 1,00 | 0,96 | 0,93 | 1,00 | 0,99 | 0,96 | 1,02 |
| **Fat percentage** | ITT-E | 130 | 1,02 | 0,99 | 1,06 | 1,04 | 0,98 | 1,12 | 1,02 | 0,96 | 1,09 |
| **Fat percentage** | PP | 120 | 1,03 | 0,99 | 1,06 | 1,05 | 0,98 | 1,12 | 1,02 | 0,96 | 1,09 |
| **HOMA-IR** | ITT-E | 130 | 1,09 | 0,85 | 1,41 | 1,33 | 0,83 | 2,14 | 1,22 | 0,79 | 1,88 |
| **HOMA-IR** | PP | 120 | 1,08 | 0,84 | 1,39 | 1,37 | 0,85 | 2,19 | 1,27 | 0,82 | 1,95 |
| **Fibrocap*** | ITT-E | 130 | -12 | -31,39 | 7,38 | -3,32 | -37,80 | 31,15 | 8,68 | -23,26 | 40,62 |
| **Fibrocap*** | PP | 120 | -12,35 | -31,32 | 6,62 | -2,66 | -36,56 | 31,23 | 9,69 | -21,14 | 40,51 |
| **Waist** | ITT-E | 130 | 1,01 | 0,98 | 1,03 | 0,99 | 0,95 | 1,03 | 0,98 | 0,95 | 1,02 |
| **Waist** | PP | 120 | 1,00 | 0,98 | 1,03 | 0,99 | 0,95 | 1,03 | 0,99 | 0,95 | 1,02 |
| **BMI** | ITT-E | 130 | 0,99 | 0,97 | 1,01 | 0,98 | 0,94 | 1,02 | 0,99 | 0,94 | 1,03 |
| **BMI** | PP | 120 | 0,99 | 0,97 | 1,01 | 0,98 | 0,94 | 1,02 | 0,99 | 0,95 | 1,03 |
| **ALT** | ITT-E | 130 | 1,14 | 0,98 | 1,32 | 1,17 | 0,88 | 1,55 | 1,03 | 0,79 | 1,33 |
| **ALT** | PP | 120 | 1,13 | 0,98 | 1,31 | 1,16 | 0,88 | 1,54 | 1,03 | 0,80 | 1,33 |
| **AST** | ITT-E | 130 | 1,06 | 0,92 | 1,23 | 1,07 | 0,83 | 1,39 | 1,01 | 0,79 | 1,30 |
| **AST** | PP | 120 | 1,07 | 0,91 | 1,24 | 1,07 | 0,83 | 1,39 | 1,01 | 0,78 | 1,30 |
| **ALT/AST ratio** | ITT-E | 130 | 1,05 | 0,87 | 1,26 | 0,97 | 0,73 | 1,29 | 0,93 | 0,71 | 1,21 |
| **ALT/AST ratio** | PP | 120 | 1,04 | 0,87 | 1,24 | 0,99 | 0,75 | 1,30 | 0,95 | 0,73 | 1,23 |
| **Insulin** | ITT-E | 130 | 0,94 | 0,76 | 1,16 | 1,14 | 0,77 | 1,70 | 1,22 | 0,85 | 1,75 |
| **Insulin** | PP | 120 | 0,94 | 0,76 | 1,16 | 1,17 | 0,79 | 1,73 | 1,24 | 0,86 | 1,79 |
| **Glucose** | ITT-E | 130 | 1,06 | 0,99 | 1,14 | 1,10 | 0,97 | 1,26 | 1,04 | 0,92 | 1,17 |
| **Glucose** | PP | 120 | 1,07 | 0,99 | 1,14 | 1,10 | 0,97 | 1,26 | 1,04 | 0,91 | 1,17 |
| **Triglycerids** | ITT-E | 130 | 0,91 | 0,77 | 1,08 | 0,83 | 0,60 | 1,13 | 0,90 | 0,68 | 1,21 |
| **Triglycerids** | PP | 120 | 0,94 | 0,80 | 1,10 | 0,84 | 0,62 | 1,14 | 0,90 | 0,68 | 1,19 |
| **Cholesterol** | ITT-E | 130 | 0,97 | 0,91 | 1,04 | 0,94 | 0,83 | 1,06 | 0,96 | 0,86 | 1,08 |
| **Cholesterol** | PP | 120 | 0,97 | 0,92 | 1,04 | 0,94 | 0,83 | 1,06 | 0,96 | 0,86 | 1,07 |
| **LDL-cholesterol** | ITT-E | 130 | 0,95 | 0,85 | 1,06 | 0,88 | 0,72 | 1,08 | 0,93 | 0,77 | 1,12 |
| **LDL-cholesterol** | PP | 120 | 0,95 | 0,86 | 1,05 | 0,88 | 0,72 | 1,07 | 0,93 | 0,77 | 1,11 |
| **HDL-cholesterol** | ITT-E | 130 | 0,95 | 0,90 | 1,01 | 0,97 | 0,87 | 1,09 | 1,02 | 0,92 | 1,13 |
| **HDL-cholesterol** | PP | 120 | 0,95 | 0,90 | 1,01 | 0,98 | 0,87 | 1,09 | 1,03 | 0,93 | 1,13 |
| **HBa1c percentage** | ITT-E | 130 | 1,00 | 0,95 | 1,04 | 0,98 | 0,91 | 1,06 | 0,99 | 0,92 | 1,06 |
| **HBa1c percentage** | PP | 120 | 1,00 | 0,96 | 1,04 | 0,98 | 0,91 | 1,05 | 0,98 | 0,92 | 1,04 |
| **HBa1c (mmol)** | ITT-E | 130 | 0,98 | 0,92 | 1,06 | 0,96 | 0,85 | 1,09 | 0,98 | 0,88 | 1,10 |
| **HBa1c (mmol)** | PP | 120 | 0,99 | 0,93 | 1,05 | 0,96 | 0,85 | 1,08 | 0,97 | 0,87 | 1,08 |
| **Limb lean mass*** | ITT-E | 130 | -140,31 | -1231,12 | 950,50 | -398,69 | -2418,65 | 1621,26 | -258,38 | -2082,97 | 1566,20 |
| **Limb lean mass*** | PP | 120 | -157,95 | -1097,00 | 781,09 | -211,59 | -1924,53 | 1501,35 | -53,64 | -1594,33 | 1487,06 |
| **Trunk fat mass** | ITT-E | 130 | 1,03 | 0,96 | 1,10 | 1,03 | 0,91 | 1,17 | 1,00 | 0,89 | 1,13 |
| **Trunk fat mass** | PP | 120 | 1,03 | 0,97 | 1,10 | 1,03 | 0,92 | 1,16 | 1,00 | 0,90 | 1,12 |
| **Limb fat mass** | ITT-E | 130 | 1,00 | 0,95 | 1,06 | 1,05 | 0,94 | 1,16 | 1,05 | 0,95 | 1,15 |
| **Limb fat mass** | PP | 120 | 1,00 | 0,95 | 1,05 | 1,05 | 0,95 | 1,16 | 1,05 | 0,95 | 1,15 |
| **Fibrolsm** | ITT-E | 130 | 0,93 | 0,82 | 1,07 | 1,25 | 0,96 | 1,62 | 1,34 | 1,06 | 1,69 |
| **Fibrolsm** | PP | 120 | 0,93 | 0,81 | 1,06 | 1,22 | 0,95 | 1,58 | 1,32 | 1,04 | 1,67 |
| **Total fat mass** | ITT-E | 130 | 0,94 | 0,87 | 1,01 | 1,08 | 0,94 | 1,25 | 1,15 | 1,01 | 1,32 |
| **Total fat mass** | PP | 120 | 0,94 | 0,88 | 1,01 | 1,08 | 0,94 | 1,24 | 1,15 | 1,01 | 1,31 |
| **AppenLeanheight²** | ITT-E | 130 | 0,99 | 0,97 | 1,00 | 0,99 | 0,96 | 1,02 | 1,00 | 0,97 | 1,03 |
| **AppenLeanheight²** | PP | 120 | 0,99 | 0,97 | 1,00 | 0,99 | 0,96 | 1,02 | 1,00 | 0,97 | 1,03 |
| **EstVFAT_MASS** | ITT-E | 130 | 1,03 | 0,96 | 1,11 | 1,01 | 0,88 | 1,17 | 0,98 | 0,86 | 1,12 |
| **EstVFAT_MASS** | PP | 120 | 1,03 | 0,96 | 1,11 | 1,02 | 0,89 | 1,17 | 0,99 | 0,87 | 1,12 |
| **Androidgynoidratio*** | ITT-E | 130 | 0,02 | -0,01 | 0,05 | 0,04 | -0,02 | 0,09 | 0,02 | -0,03 | 0,06 |
| **Androidgynoidratio*** | PP | 120 | 0,02 | 0,00 | 0,05 | 0,04 | -0,01 | 0,09 | 0,02 | -0,03 | 0,06 |
| **Body lean mass** | ITT-E | 130 | 0,98 | 0,97 | 1,00 | 0,98 | 0,95 | 1,01 | 1,00 | 0,97 | 1,03 |
| **Body lean mass** | PP | 120 | 0,98 | 0,97 | 1,00 | 0,98 | 0,95 | 1,01 | 0,99 | 0,97 | 1,02 |
| **Systolic blood pressure** | ITT-E | 130 | 1,02 | 0,98 | 1,06 | 1,03 | 0,96 | 1,11 | 1,01 | 0,94 | 1,08 |
| **Systolic blood pressure** | PP | 120 | 1,02 | 0,98 | 1,06 | 1,03 | 0,96 | 1,11 | 1,01 | 0,94 | 1,08 |
| **Diastolic blood pressure** | ITT-E | 130 | 1,03 | 0,99 | 1,07 | 1,06 | 0,98 | 1,15 | 1,04 | 0,96 | 1,11 |
| **Diastolic blood pressure** | PP | 120 | 1,03 | 0,99 | 1,07 | 1,07 | 0,99 | 1,15 | 1,04 | 0,97 | 1,11 |

Metabolic changes after 48 weeks, adjusted for baseline response value, baseline regimen and baseline BMI in the ITT-E (n=130) and Per Protocol (PP) population (n=120). Estimated ratios are reported with upper and lower confidence interval limit estimates, calculated using an ordinary linear regression model applied to change from baseline in natural log-transformed imputed data.

*For untransformed endpoints, the estimated arithmetic mean difference in (absolute) change from baseline between groups is reported with 95% Wald CI.

Per Protocol set: Patients allocated to 2DR, who switch to or stay on 3DR (did not reach W48) are excluded from the per protocol analysis (DTG/3TC=6, B/F/TAF=3). In case of a recurrent viral load above 50 copies/ml or in case of a single viral load measurement above 200 copies/ml (DTG/3TC=1), the patient is also excluded from the per protocol population.

**Figure S2: gating strategy
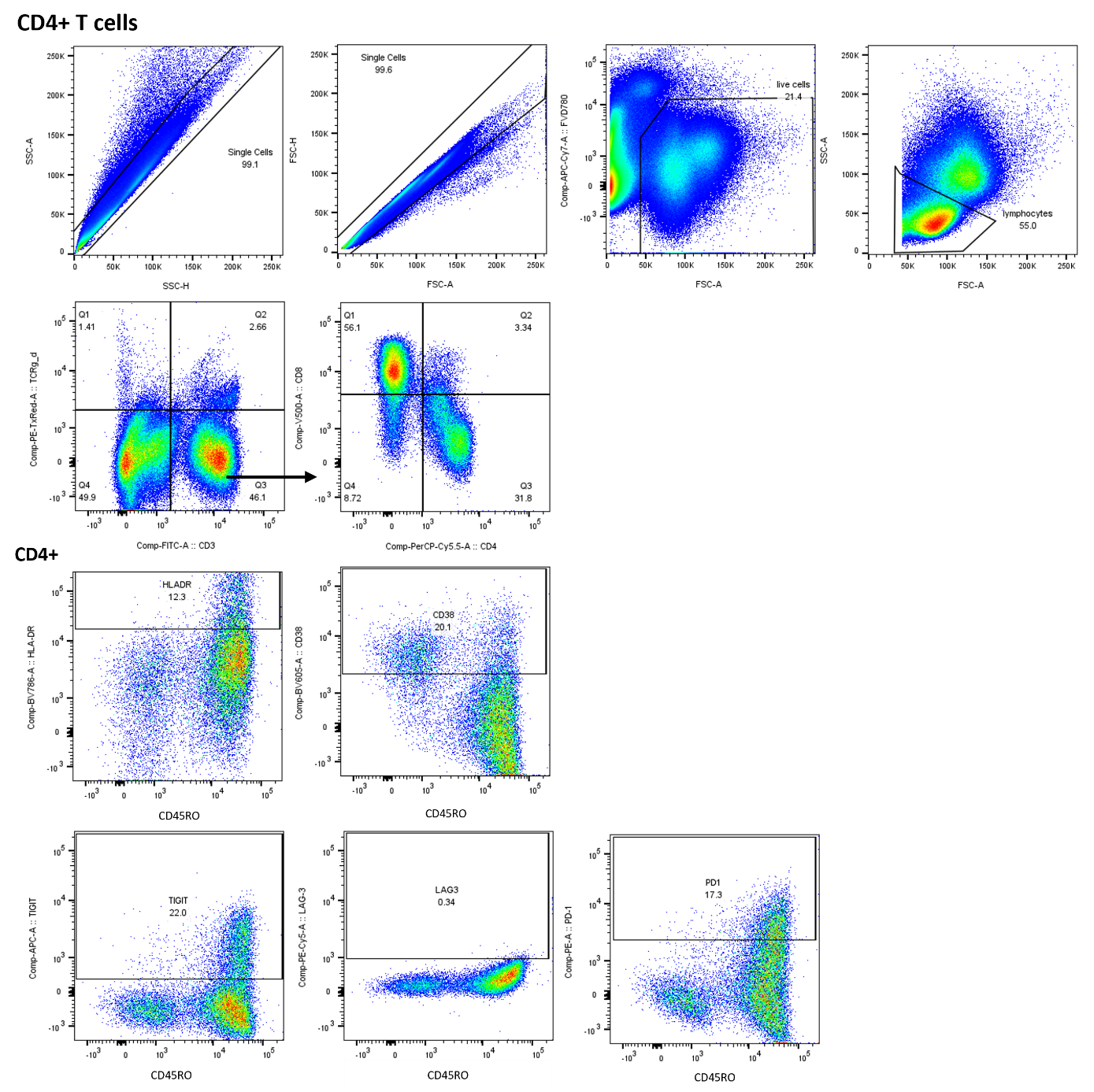
**

**
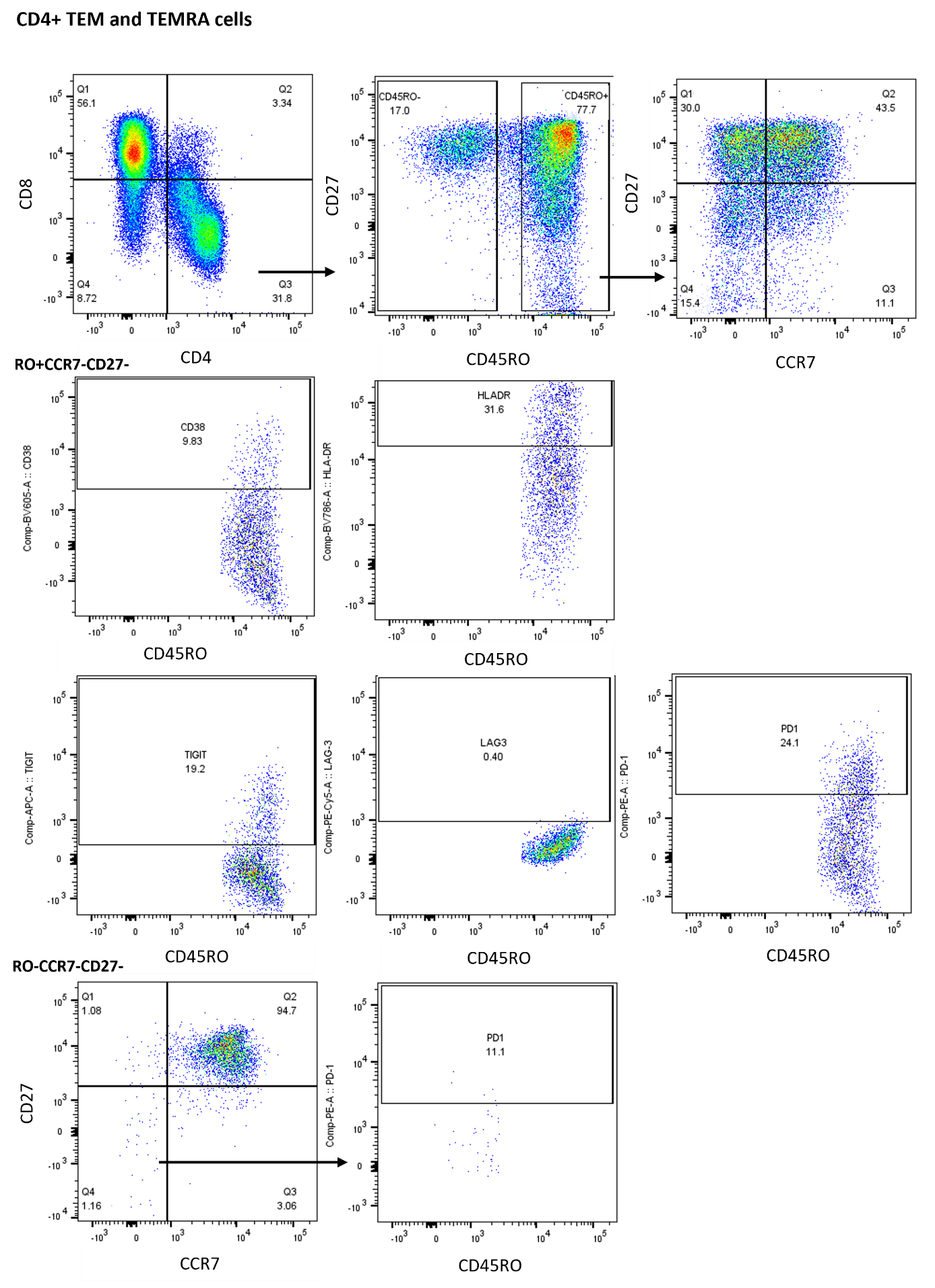

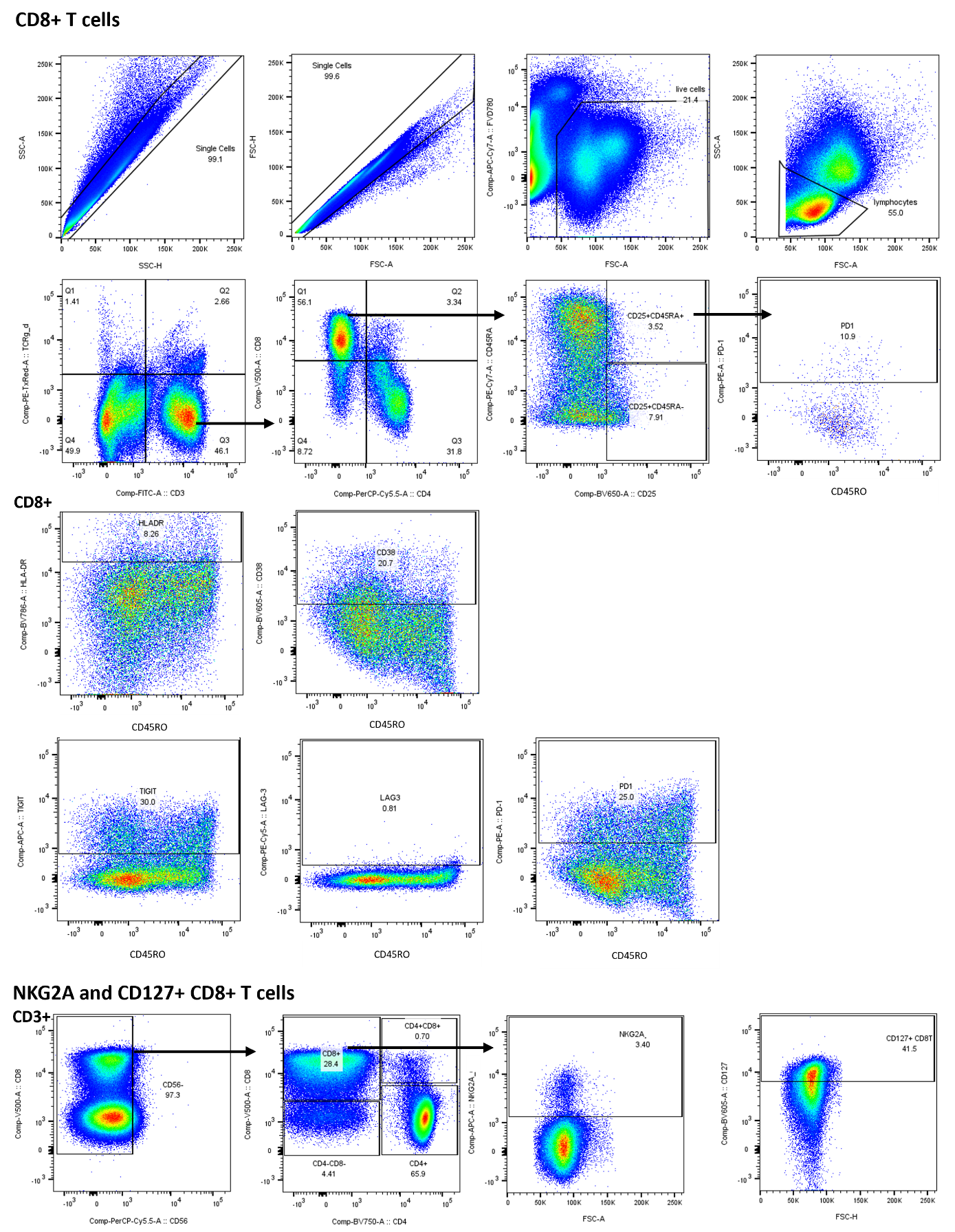

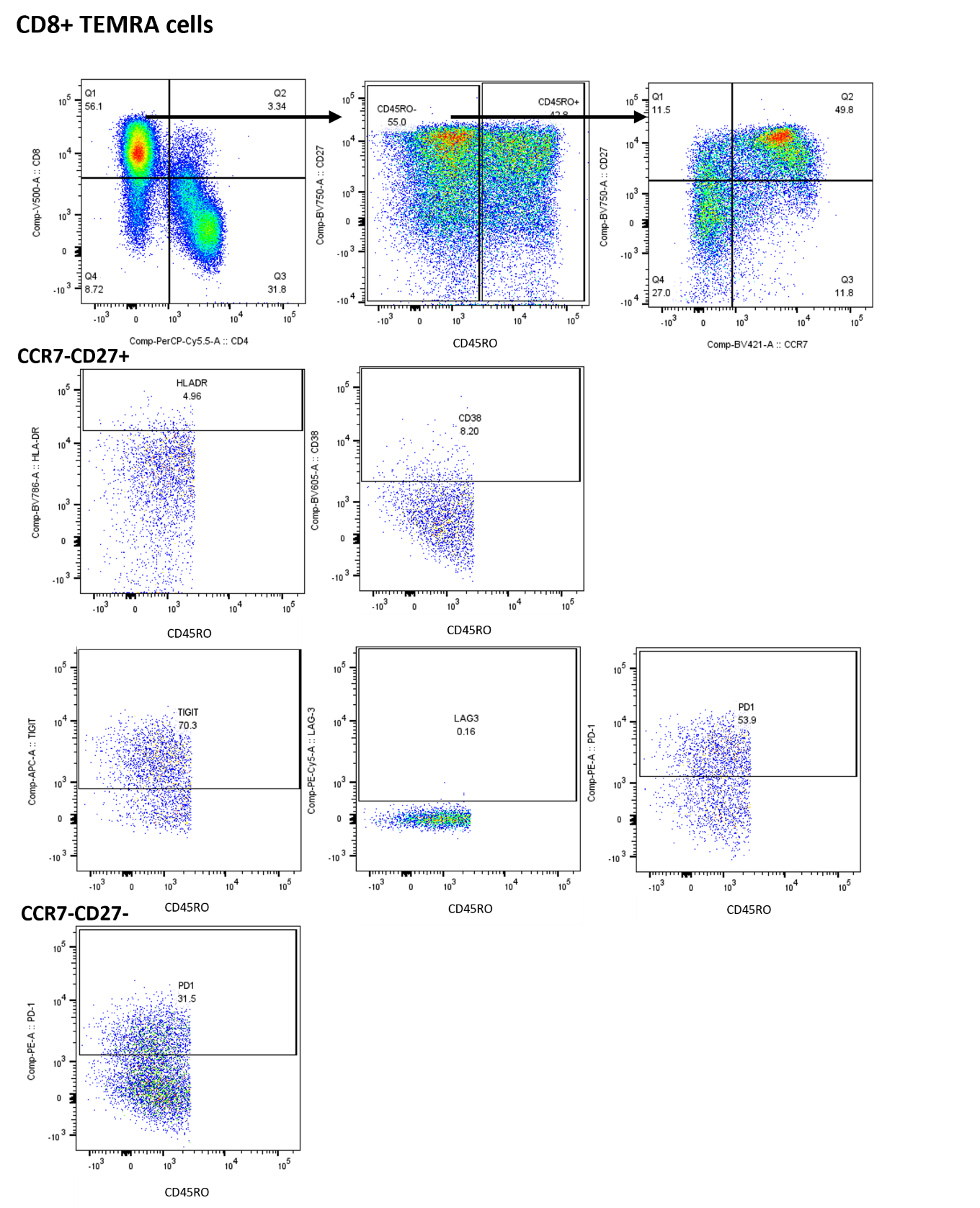
**

**
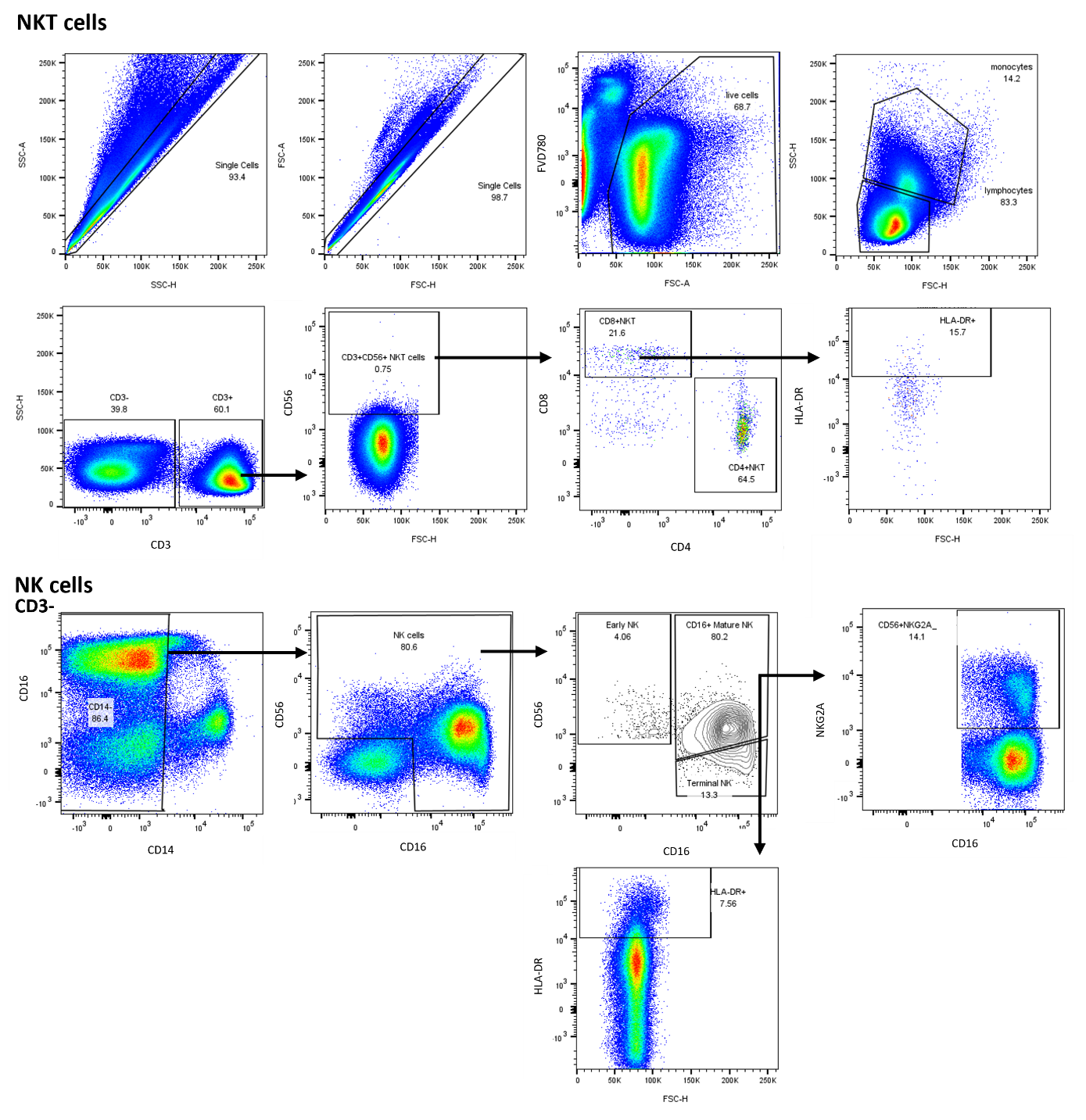

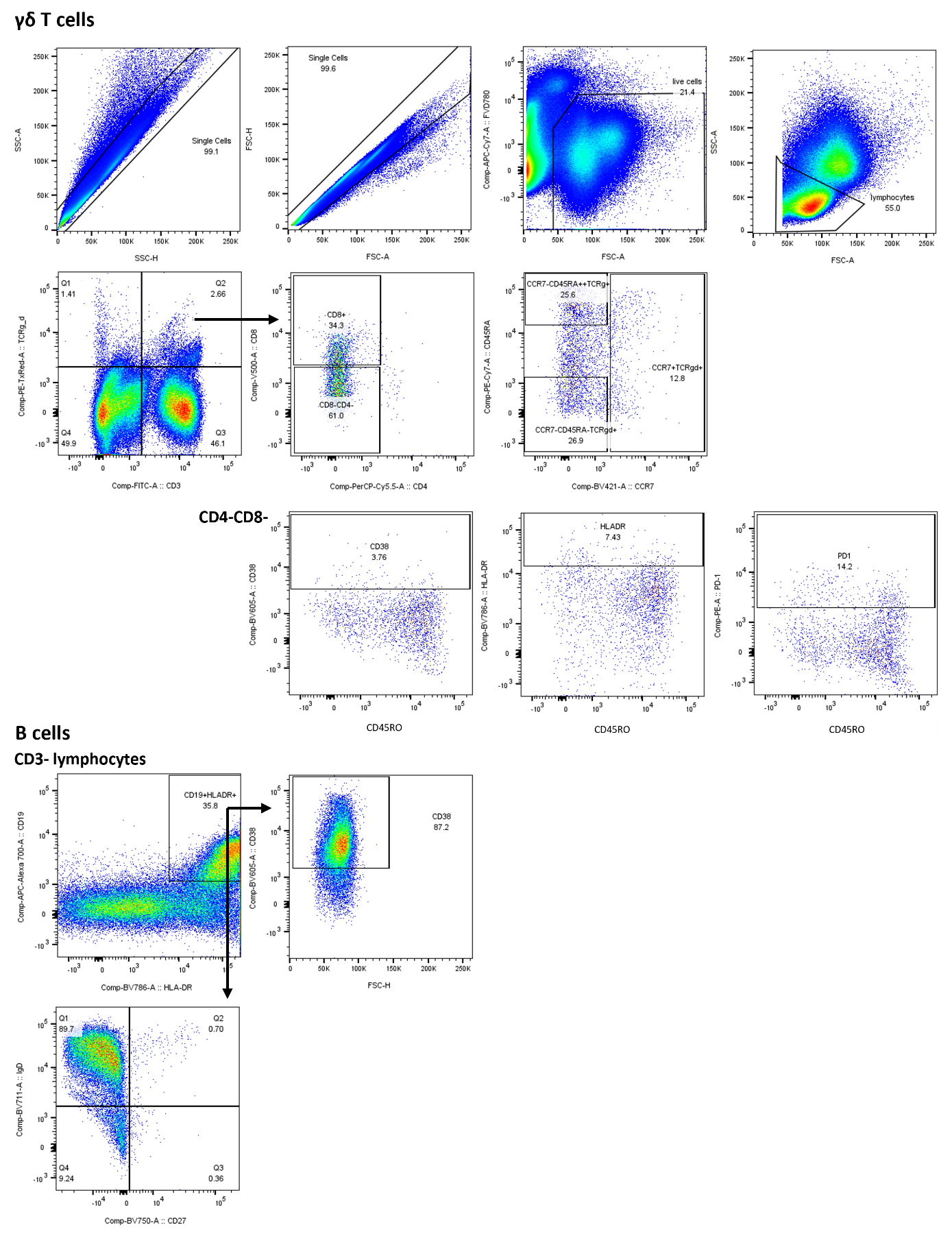
**

**
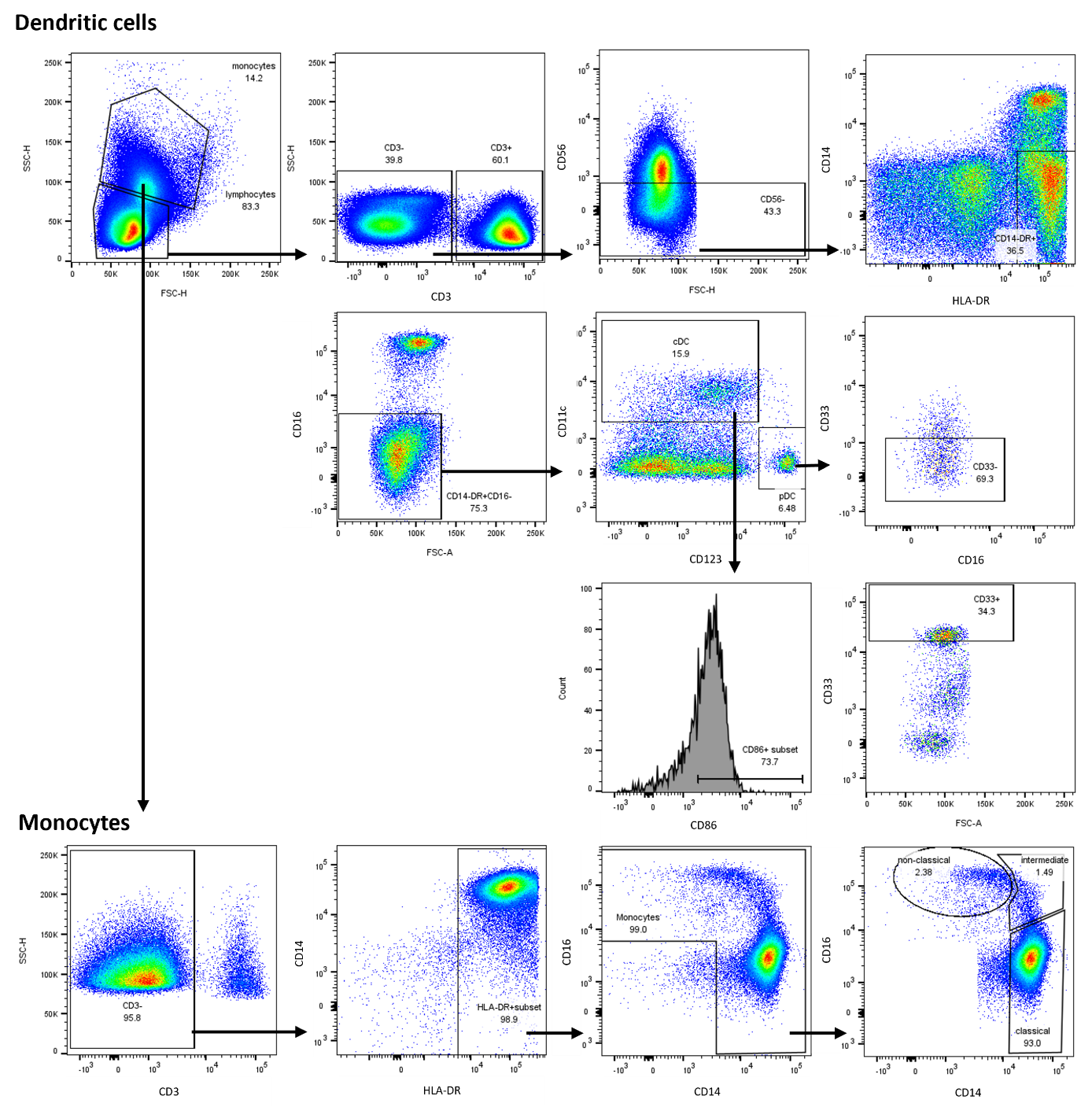
**

**References supplementary file 1**

1. Cassidy NAJ, Fish CS, Levy CN, et al. HIV reservoir quantification using cross-subtype multiplex ddPCR. iScience 2022; 25(1): 103615.

2. Bruner KM, Wang Z, Simonetti FR, et al. A quantitative approach for measuring the reservoir of latent HIV-1 proviruses. Nature 2019; 566(7742): 120-5.

3. Yukl SA, Kaiser P, Kim P, et al. HIV latency in isolated patient CD4(+) T cells may be due to blocks in HIV transcriptional elongation, completion, and splicing. Science translational medicine 2018; 10(430).

4. Batterham RL, Espinosa N, Katlama C, et al. Cardiometabolic Parameters 3 Years After Switch to Dolutegravir/Lamivudine vs Maintenance of Tenofovir Alafenamide-Based Regimens. Open forum infectious diseases 2023; 10(7): ofad359.

5. Barcelo H, Faul J, Crimmins E, Thyagarajan B. A Practical Cryopreservation and Staining Protocol for Immunophenotyping in Population Studies. Current protocols in cytometry 2018; 84(1): e35.

6. Emmaneel A, Quintelier K, Sichien D, et al. PeacoQC: Peak-based selection of high quality cytometry data. Cytometry Part A : the journal of the International Society for Analytical Cytology 2022; 101(4): 325-38.

7. Yun Z, Fredriksson E, Sonnerborg A. Quantification of human immunodeficiency virus type 1 proviral DNA by the TaqMan real-time PCR assay. J Clin Microbiol 2002; 40(10): 3883-4.

8. Yu JJ, Wu TL, Liszewski MK, et al. A more precise HIV integration assay designed to detect small differences finds lower levels of integrated DNA in HAART treated patients. Virology 2008; 379(1): 78-86.

# Supplementary file 2: Complete case analyses

1. **Virological endpoints**

|  |  |  | **ITT-E N=130** | | | **B/F/TAF (3DR)** | | | **3TC/DTG (2DR)** | | |
| --- | --- | --- | --- | --- | --- | --- | --- | --- | --- | --- | --- |
|  |  |  | **Treatment ratio 3DR/2DR** | | | **W48 visit to baseline ratio** | | | **W48 visit to baseline ratio** | | |
| **Endpoint** | **Population** | **N** | **Estimate** | **95% LCL** | **95% UCL** | **Estimate** | **95% LCL** | **95% UCL** | **Estimate** | **95% LCL** | **95% UCL** |
| **Intact HIV-1 DNA** | ITT-E | 96 | 1,45 | 0,90 | 2,35 | 1,36 | 0,91 | 2,04 | 0,94 | 0,73 | 1,21 |
| **Intact HIV-1 DNA** | PP | 96 | 1,45 | 0,90 | 2,35 | 1,36 | 0,91 | 2,04 | 0,94 | 0,73 | 1,21 |
| **Intact HIV-1 DNA*** | ITT-E | 88 | 1,26 | 0,74 | 2,14 | 0,56 | 0,25 | 1,28 | 0,45 | 0,22 | 0,93 |
| **Intact HIV-1 DNA**** | ITT-E | 96 | 1,48 | 0,92 | 2,38 | 1,38 | 0,93 | 2,05 | 0,93 | 0,73 | 1,20 |
| **Intact HIV-1 DNA without DSI** | ITT-E | 96 | 1,48 | 0,92 | 2,38 | 1,41 | 0,95 | 2,10 | 0,95 | 0,74 | 1,23 |
| **Intact HIV-1 DNA without DSI** | PP | 96 | 1,48 | 0,92 | 2,38 | 1,41 | 0,95 | 2,10 | 0,95 | 0,74 | 1,23 |
| **Intact HIV-1 DNA without DSI*** | ITT-E | 88 | 1,29 | 0,76 | 2,19 | 0,61 | 0,27 | 1,37 | 0,47 | 0,23 | 0,97 |

**2. Immune activation and exhaustion markers**

|  |  | **ITT-E N=130** | |  | **B/F/TAF (3DR)** | | |  | **3TC/DTG (2DR)** | | |  |  |
| --- | --- | --- | --- | --- | --- | --- | --- | --- | --- | --- | --- | --- | --- |
|  |  | **Treatment ratio 3DR/2DR** | | | | **W48 visit to baseline ratio** | | | | **W48 visit to baseline ratio** | | | |
| **Endpoint** | **N** | **Estimate** | **95% LCL** | **95% UCL** | **Estimate** | | **95% LCL** | **95% UCL** | **Estimate** | | **95% LCL** | **95% UCL** |  |
| **CD4 T** | 113 | -1,07 | -2,87 | 0,72 | 0,23 | | -1,33 | 1,8 | 1,31 | | 0,24 | 2,37 |  |
| **HLA-DR** | 113 | 1,08 | 0,98 | 1,2 | 1,11 | | 1,01 | 1,21 | 1,02 | | 0,96 | 1,08 |  |
| **CD38*** | 113 | 0,95 | -0,85 | 2,75 | 0,95 | | -0,63 | 2,53 | 0,00 | | -1,09 | 1,08 |  |
| **TIGIT** | 113 | 0,96 | 0,92 | 1,01 | 1,00 | | 0,96 | 1,04 | 1,04 | | 1,01 | 1,07 |  |
| **LAG-3** | 113 | 0,85 | 0,66 | 1,08 | 1,00 | | 0,81 | 1,24 | 1,18 | | 1,02 | 1,37 |  |
| **PD-1** | 113 | 0,99 | 0,91 | 1,08 | 1,04 | | 0,96 | 1,12 | 1,05 | | 1,00 | 1,11 |  |
| **TEM** | 113 | 1,05 | 0,95 | 1,15 | 1,02 | | 0,94 | 1,1 | 0,97 | | 0,92 | 1,03 |  |
| **HLA-DR** | 113 | 1,19 | 1,05 | 1,36 | 1,18 | | 1,06 | 1,32 | 0,99 | | 0,92 | 1,07 |  |
| **HLA-DR+ PD-1+ CD38-** | 113 | 1,18 | 1,03 | 1,36 | 1,14 | | 1,01 | 1,28 | 0,96 | | 0,89 | 1,04 |  |
| **TEMRA** | 113 | 1,23 | 0,96 | 1,58 | 1,15 | | 0,92 | 1,43 | 0,94 | | 0,81 | 1,09 |  |
| **PD-1** | 113 | 0,79 | 0,65 | 0,94 | 0,89 | | 0,76 | 1,05 | 1,13 | | 1,02 | 1,26 |  |
| **CD8 T** | 113 | 1,02 | 0,97 | 1,07 | 1,00 | | 0,96 | 1,04 | 0,98 | | 0,95 | 1,01 |  |
| **HLA-DR** | 113 | 1,08 | 0,91 | 1,29 | 1,23 | | 1,05 | 1,44 | 1,14 | | 1,02 | 1,26 |  |
| **CD38** | 113 | 1,08 | 0,97 | 1,2 | 1,1 | | 1,01 | 1,21 | 1,02 | | 0,96 | 1,09 |  |
| **TIGIT** | 113 | 0,98 | 0,93 | 1,03 | 1,02 | | 0,97 | 1,07 | 1,04 | | 1,01 | 1,07 |  |
| **LAG-3** | 113 | 0,85 | 0,69 | 1,05 | 1,08 | | 0,9 | 1,3 | 1,27 | | 1,12 | 1,44 |  |
| **PD-1** | 113 | 0,94 | 0,88 | 1,00 | 1,02 | | 0,96 | 1,08 | 1,08 | | 1,04 | 1,13 |  |
| **PD-1+ HLA-DR- CD38-** | 113 | 0,9 | 0,84 | 0,97 | 0,96 | | 0,91 | 1,02 | 1,07 | | 1,03 | 1,11 |  |
| **CD27+ TEMRA** | 113 | 1,01 | 0,92 | 1,11 | 1,00 | | 0,93 | 1,08 | 0,99 | | 0,94 | 1,05 |  |
| **PD-1** | 113 | 0,92 | 0,87 | 0,99 | 0,98 | | 0,93 | 1,04 | 1,06 | | 1,02 | 1,1 |  |
| **PD-1+ HLA-DR- CD38-** | 113 | 0,87 | 0,77 | 0,97 | 0,97 | | 0,88 | 1,08 | 1,12 | | 1,04 | 1,2 |  |
| **TIGIT** | 113 | 0,93 | 0,88 | 0,98 | 0,97 | | 0,93 | 1,01 | 1,05 | | 1,02 | 1,08 |  |
| **CD27- TEMRA** | 113 | 1,05 | 0,93 | 1,19 | 0,98 | | 0,88 | 1,09 | 0,92 | | 0,86 | 1,00 |  |
| **PD-1+ HLA-DR- CD38-** | 113 | 0,84 | 0,73 | 0,97 | 0,92 | | 0,81 | 1,04 | 1,09 | | 1,01 | 1,19 |  |
| **CD25+ CD45RA+** | 113 | 1,09 | 0,96 | 1,24 | 1,01 | | 0,9 | 1,12 | 0,92 | | 0,86 | 1,00 |  |
| **PD-1** | 113 | 0,88 | 0,8 | 0,97 | 0,95 | | 0,88 | 1,04 | 1,08 | | 1,02 | 1,15 |  |
| **CD8 NKT** | 113 | 0,97 | 0,88 | 1,08 | 0,96 | | 0,88 | 1,05 | 0,99 | | 0,93 | 1,05 |  |
| **HLA-DR** | 113 | 1,68 | 1,19 | 2,36 | 1,58 | | 1,17 | 2,13 | 0,94 | | 0,77 | 1,15 |  |
| **γδ T** | 112 | 0,89 | 0,81 | 0,98 | 0,91 | | 0,83 | 0,99 | 1,01 | | 0,96 | 1,07 |  |
| **CD8+** | 112 | 1,1 | 1,03 | 1,18 | 1,05 | | 0,99 | 1,11 | 0,95 | | 0,92 | 0,99 |  |
| **CD4-CD8-** | 112 | 0,9 | 0,83 | 0,96 | 0,94 | | 0,88 | 1,00 | 1,05 | | 1,01 | 1,1 |  |
| **HLA-DR** | 112 | 1,16 | 0,96 | 1,4 | 1,3 | | 1,1 | 1,53 | 1,12 | | 1,00 | 1,25 |  |
| **CD38** | 112 | 1,29 | 1,09 | 1,54 | 1,25 | | 1,07 | 1,46 | 0,97 | | 0,87 | 1,07 |  |
| **PD-1** | 112 | 1,38 | 1,25 | 1,53 | 1,21 | | 1,1 | 1,32 | 0,87 | | 0,82 | 0,92 |  |
| **LAG-3 (MFI)*** | 112 | -4,18 | -14,76 | 6,41 | 0,45 | | -8,86 | 9,77 | 4,63 | | -1,63 | 10,89 |  |
| **TIGIT (MFI)** | 112 | 1,93 | 1,43 | 2,6 | 1,59 | | 1,23 | 2,07 | 0,83 | | 0,69 | 0,99 |  |

1. **Secondary and exploratory metabolic outcomes**

|  |  |  | **ITT-E N=130** | | | **B/F/TAF (3DR)** | | | **3TC/DTG (2DR)** | | |
| --- | --- | --- | --- | --- | --- | --- | --- | --- | --- | --- | --- |
|  |  |  | Treatment ratio 3DR/2DR | | | W48 visit to baseline ratio | | | W48 visit to baseline ratio | | |
| **Endpoint** | **Population** | **N** | **Estimate** | **95% LCL** | **95% UCL** | **Estimate** | **95% LCL** | **95% UCL** | **Estimate** | **95% LCL** | **95% UCL** |
| **Weight** | ITT-E | 118 | 0,99 | 0,97 | 1,01 | 0,98 | 0,94 | 1,02 | 0,98 | 0,95 | 1,02 |
| **Weight** | PP | 117 | 0,99 | 0,97 | 1,01 | 0,98 | 0,94 | 1,02 | 0,98 | 0,95 | 1,02 |
| **Cholesterol/HDL ratio** | ITT-E | 109 | 1,01 | 0,94 | 1,09 | 0,97 | 0,85 | 1,10 | 0,95 | 0,85 | 1,07 |
| **Cholesterol/HDL ratio** | PP | 108 | 1,01 | 0,94 | 1,09 | 0,97 | 0,85 | 1,10 | 0,95 | 0,85 | 1,07 |
| **Trunk lean mass** | ITT-E | 117 | 0,97 | 0,96 | 0,99 | 0,96 | 0,93 | 1,00 | 0,99 | 0,96 | 1,02 |
| **Trunk lean mass** | PP | 116 | 0,98 | 0,96 | 0,99 | 0,96 | 0,93 | 1,00 | 0,99 | 0,96 | 1,02 |
| **Fat percentage** | ITT-E | 117 | 1,03 | 1,00 | 1,07 | 1,05 | 0,99 | 1,12 | 1,02 | 0,96 | 1,08 |
| **Fat percentage** | PP | 116 | 1,03 | 1,00 | 1,07 | 1,05 | 0,99 | 1,12 | 1,02 | 0,96 | 1,08 |
| **HOMA-IR** | ITT-E | 92 | 0,92 | 0,75 | 1,13 | 1,13 | 0,78 | 1,64 | 1,23 | 0,87 | 1,73 |
| **HOMA-IR** | PP | 92 | 0,92 | 0,75 | 1,13 | 1,13 | 0,78 | 1,64 | 1,23 | 0,87 | 1,73 |
| **Fibrocap*** | ITT-E | 92 | -13,07 | -30,34 | 4,2 | -6,13 | -36,36 | 24,09 | 6,94 | -20,38 | 34,25 |
| **Fibrocap*** | PP | 91 | -13,41 | -30,80 | 3,98 | -6,68 | -37,04 | 23,69 | 6,73 | -20,69 | 34,16 |
| **Waist** | ITT-E | 117 | 1,00 | 0,98 | 1,02 | 0,99 | 0,96 | 1,03 | 0,99 | 0,96 | 1,02 |
| **Waist** | PP | 116 | 1,00 | 0,98 | 1,02 | 0,99 | 0,96 | 1,03 | 0,99 | 0,96 | 1,02 |
| **BMI** | ITT-E | 118 | 0,99 | 0,97 | 1,01 | 0,98 | 0,94 | 1,02 | 0,99 | 0,95 | 1,03 |
| **BMI** | PP | 117 | 0,99 | 0,97 | 1,01 | 0,98 | 0,94 | 1,02 | 0,99 | 0,95 | 1,03 |
| **ALT** | ITT-E | 68 | 1,08 | 0,88 | 1,33 | 1,1 | 0,79 | 1,52 | 1,01 | 0,76 | 1,35 |
| **ALT** | PP | 68 | 1,08 | 0,88 | 1,33 | 1,1 | 0,79 | 1,52 | 1,01 | 0,76 | 1,35 |
| **AST** | ITT-E | 35 | 1,19 | 0,99 | 1,41 | 1,13 | 1,00 | 1,27 | 0,95 | 0,85 | 1,07 |
| **AST** | PP | 35 | 1,19 | 0,99 | 1,41 | 1,13 | 1,00 | 1,27 | 0,95 | 0,85 | 1,07 |
| **ALT/AST ratio** | ITT-E | 35 | 1,00 | 0,81 | 1,24 | 0,96 | 0,82 | 1,11 | 0,96 | 0,83 | 1,10 |
| **ALT/AST ratio** | PP | 35 | 1,00 | 0,81 | 1,24 | 0,96 | 0,82 | 1,11 | 0,96 | 0,83 | 1,10 |
| **Insulin** | ITT-E | 96 | 0,89 | 0,72 | 1,09 | 1,05 | 0,72 | 1,53 | 1,19 | 0,84 | 1,68 |
| **Insulin** | PP | 96 | 0,89 | 0,72 | 1,09 | 1,05 | 0,72 | 1,53 | 1,19 | 0,84 | 1,68 |
| **Glucose** | ITT-E | 100 | 1,06 | 0,99 | 1,14 | 1,10 | 0,96 | 1,25 | 1,03 | 0,91 | 1,17 |
| **Glucose** | PP | 100 | 1,06 | 0,99 | 1,14 | 1,10 | 0,96 | 1,25 | 1,03 | 0,91 | 1,17 |
| **Triglycerids** | ITT-E | 106 | 0,92 | 0,79 | 1,08 | 0,84 | 0,63 | 1,13 | 0,92 | 0,70 | 1,2 |
| **Triglycerids** | PP | 105 | 0,93 | 0,79 | 1,09 | 0,85 | 0,63 | 1,14 | 0,92 | 0,70 | 1,2 |
| **Cholesterol** | ITT-E | 111 | 0,97 | 0,91 | 1,03 | 0,94 | 0,83 | 1,05 | 0,97 | 0,87 | 1,08 |
| **Cholesterol** | PP | 110 | 0,97 | 0,91 | 1,03 | 0,94 | 0,84 | 1,05 | 0,97 | 0,87 | 1,08 |
| **LDL-cholesterol** | ITT-E | 105 | 0,93 | 0,84 | 1,04 | 0,88 | 0,72 | 1,07 | 0,94 | 0,78 | 1,13 |
| **LDL-cholesterol** | PP | 104 | 0,93 | 0,84 | 1,04 | 0,88 | 0,72 | 1,07 | 0,94 | 0,78 | 1,13 |
| **HDL-cholesterol** | ITT-E | 110 | 0,96 | 0,9 | 1,01 | 0,98 | 0,88 | 1,09 | 1,02 | 0,92 | 1,13 |
| **HDL-cholesterol** | PP | 109 | 0,96 | 0,9 | 1,01 | 0,98 | 0,87 | 1,09 | 1,02 | 0,92 | 1,13 |
| **HBa1c percentage** | ITT-E | 31 | 0,99 | 0,96 | 1,02 | 0,98 | 0,95 | 1,00 | 0,99 | 0,97 | 1,00 |
| **HBa1c percentage** | PP | 31 | 0,99 | 0,96 | 1,02 | 0,98 | 0,95 | 1,00 | 0,99 | 0,97 | 1,00 |
| **HBa1c (mmol)** | ITT-E | 32 | 0,99 | 0,94 | 1,04 | 0,98 | 0,94 | 1,02 | 0,99 | 0,96 | 1,01 |
| **HBa1c (mmol)** | PP | 32 | 0,99 | 0,94 | 1,04 | 0,98 | 0,94 | 1,02 | 0,99 | 0,96 | 1,01 |
| **Limb lean mass*** | ITT-E | 106 | -340,15 | -943,2 | 262,9 | -503,1 | -1615,6 | 609,4 | -162,95 | -1162,9 | 837 |
| **Limb lean mass*** | PP | 106 | -340,15 | -943,2 | 262,9 | -503,1 | -1615,6 | 609,4 | -162,95 | -1162,9 | 837 |
| **Trunk fat mass** | ITT-E | 117 | 1,03 | 0,97 | 1,10 | 1,03 | 0,92 | 1,16 | 1,00 | 0,9 | 1,12 |
| **Trunk fat mass** | PP | 116 | 1,03 | 0,97 | 1,10 | 1,03 | 0,92 | 1,16 | 1,00 | 0,9 | 1,12 |
| **Limb fat mass** | ITT-E | 118 | 1,00 | 0,95 | 1,05 | 1,04 | 0,95 | 1,14 | 1,04 | 0,95 | 1,14 |
| **Limb fat mass** | PP | 117 | 1,00 | 0,95 | 1,05 | 1,04 | 0,95 | 1,15 | 1,05 | 0,96 | 1,14 |
| **Fibrolsm** | ITT-E | 116 | 0,95 | 0,83 | 1,08 | 1,22 | 0,95 | 1,57 | 1,29 | 1,03 | 1,63 |
| **Fibrolsm** | PP | 115 | 0,95 | 0,83 | 1,08 | 1,22 | 0,95 | 1,57 | 1,29 | 1,03 | 1,63 |
| **Total fat mass** | ITT-E | 118 | 0,94 | 0,88 | 1,01 | 1,08 | 0,94 | 1,24 | 1,15 | 1,01 | 1,31 |
| **Total fat mass** | PP | 117 | 0,94 | 0,88 | 1,01 | 1,09 | 0,95 | 1,25 | 1,15 | 1,01 | 1,31 |
| **AppenLeanheight²** | ITT-E | 120 | 0,99 | 0,97 | 1,00 | 0,99 | 0,96 | 1,02 | 1,00 | 0,97 | 1,03 |
| **AppenLeanheight²** | PP | 119 | 0,99 | 0,97 | 1,00 | 0,99 | 0,96 | 1,02 | 1,00 | 0,97 | 1,03 |
| **EstVFAT_MASS** | ITT-E | 120 | 1,03 | 0,96 | 1,11 | 1,02 | 0,89 | 1,17 | 0,99 | 0,87 | 1,12 |
| **EstVFAT_MASS** | PP | 119 | 1,03 | 0,96 | 1,11 | 1,02 | 0,89 | 1,17 | 0,99 | 0,87 | 1,12 |
| **Androidgynoid ratio*** | ITT-E | 120 | 0,02 | 0 | 0,05 | 0,04 | -0,01 | 0,09 | 0,02 | -0,03 | 0,06 |
| **Androidgynoid ratio*** | PP | 119 | 0,02 | 0 | 0,05 | 0,04 | -0,01 | 0,09 | 0,02 | -0,03 | 0,06 |
| **Body lean mass** | ITT-E | 119 | 0,98 | 0,97 | 1,00 | 0,98 | 0,95 | 1,01 | 0,99 | 0,97 | 1,02 |
| **Body lean mass** | PP | 118 | 0,98 | 0,97 | 1,00 | 0,98 | 0,95 | 1,01 | 0,99 | 0,97 | 1,02 |
| **Systolic blood pressure** | ITT-E | 118 | 1,02 | 0,99 | 1,06 | 1,03 | 0,96 | 1,11 | 1,01 | 0,94 | 1,08 |
| **Systolic blood pressure** | PP | 117 | 1,02 | 0,98 | 1,06 | 1,03 | 0,96 | 1,11 | 1,01 | 0,94 | 1,08 |
| **Diastolic blood pressure** | ITT-E | 118 | 1,03 | 0,99 | 1,07 | 1,07 | 0,99 | 1,16 | 1,04 | 0,97 | 1,11 |
| **Diastolic blood pressure** | PP | 117 | 1,03 | 0,99 | 1,07 | 1,07 | 0,99 | 1,16 | 1,04 | 0,97 | 1,11 |
